# Cost-effectiveness analysis of switching from a bivalent to a nonavalent HPV vaccination programme in China: a modelling study

**DOI:** 10.1016/j.lanwpc.2025.101499

**Published:** 2025-02-20

**Authors:** Meng Gao, Shangying Hu, Xuelian Zhao, Tingting You, Yuting Hong, Yang Liu, Youlin Qiao, Mark Jit, Fanghui Zhao, Chen Wang

**Affiliations:** a Department of Cancer Epidemiology, National Cancer Center/National Clinical Research Center for Cancer/Cancer Hospital, Chinese Academy of Medical Sciences and Peking Union Medical College, Beijing, China; b Department of Epidemiology, Beijing Neurosurgical Institute, Beijing Tiantan Hospital, Capital Medical University, Beijing, China; c School of Population Medicine and Public Health, Chinese Academy of Medical Sciences & Peking Union Medical College, Beijing, China; d Scientific Research Center, China-Japan Friendship Hospital, Beijing, China; e Department of Infectious Disease Epidemiology, Faculty of Epidemiology and Population Health, London School of Hygiene & Tropical Medicine, London, United Kingdom; f School of Public Health, University of Hong Kong, Hong Kong, Hong Kong SAR, China

**Keywords:** Human papillomavirus vaccination, Nonavalent, Cost-effectiveness, Elimination, Mixed schedule

## Abstract

**Background:**

Several domestically-manufactured nonavalent HPV vaccine candidates are in phase III clinical trials and their future availability may address the current dilemma of insufficient supply and high price of the overseas-manufactured nonavalent HPV vaccine in China. We compare the population-level effectiveness and cost-effectiveness of switching to nonavalent HPV vaccination in China.

**Methods:**

We used a previously validated transmission model to project the lifetime costs and effectiveness of five same-vaccine and two mixed-vaccine strategies. Nonavalent HPV vaccines were assumed to be available and meet the production requirements for national vaccination between 2030 and 2050. All women living or projected to be born in China during 2023–2100 were considered. We adopted a societal perspective and determined optimal strategies using cost-effectiveness efficiency frontiers.

**Findings:**

Under our pricing assumptions, switching to nonavalent vaccination was always cost-saving compared with maintaining the current bivalent vaccination programme, irrespective of the screening scenarios and the year when nonavalent vaccine was assumed to become available (status quo screening: net cost saving $2589–5211 million; improved screening: net cost saving $1852–3789 million). In the same-vaccine strategies, the optimal strategy changed from “routine nonavalent HPV vaccination with catch-up to age 18” to “switching from bivalent to nonavalent HPV vaccination” if nonavalent vaccination is available after 2035. Compared with the optimal same-vaccine strategy, adopting mixed schedules with bivalent and nonavalent vaccines would further save $1336–4280 million net costs and gain 87,000–833,000 QALYs, depending on the screening scenario and the year when nonavalent vaccine becomes available.

**Interpretation:**

Switching from bivalent to nonavalent HPV vaccination is likely to be cost-saving and have a significant impact on reducing the cervical cancer burden in China.

**Funding:**

10.13039/100000865Bill & Melinda Gates Foundation (INV-031449 and INV-003174) and CAMS Innovation Fund for Medical Sciences (CIFMS) (2021-I2M-1-004).

**Research in context**

**Evidence before this study**

Many high-income countries have replaced two-valent (2vHPV) or four-valent (4vHPV) HPV vaccines with the nine-valent (9vHPV) vaccine in their national immunisation programmes, partly based on modelling studies establishing the superior impact and cost-effectiveness of the 9vHPV vaccine. However, the constrained supply and high price of the 9-valent vaccine has hindered its widespread availability, especially in low- and middle-income countries (LMICs). In China, there are three brands of 2vHPV vaccine on the market providing enough supply for the national programme, but only one brand of 9vHPV with insufficient supply for this purpose. However, a mixed vaccination schedule of a dose each of 2vHPV and 9vHPV has been shown to be highly immunogenic and has been implemented in Quebec, Canada, which opens new options for utilizing supplies of 2vHPV and 9vHPV vaccines. We searched PubMed and China National Knowledge Infrastructure, without language restrictions, for studies on the cost-effectiveness of 9vHPV vaccination published from Jan 1, 2000, to June 1, 2024, with the search terms “cost-effectiveness” or “elimination”, “nonavalent” or “9-valent”, and “human papillomavirus”. Our search identified three studies comparing the cost-effectiveness of 9vHPV vaccination with 2vHPV vaccination in China. However, none of these studies use the latest recommended two-dose 9vHPV vaccination schedule for adolescent girls. Additionally, no study considered strategies that account for availability of 9vHPV, such as the potential inclusion of mixed-vaccine strategies.

**Added value of this study**

This is the first cost-effectiveness analysis of switching from 2vHPV to 9vHPV vaccination that considers scenarios about future 9vHPV vaccine availability and mixed-vaccine strategies. Our findings indicate that switching to 9vHPV vaccination was cost-saving compared to maintaining 2vHPV vaccination in China. If only vaccine schedules using the same vaccine type are possible, the optimal vaccination strategy is to wait for routine 9vHPV vaccination with a catch-up to age 18 if 9vHPV vaccine is initiated in 2030 and to initiate bivalent vaccination immediately with a switch to 9vHPV vaccination if 9vHPV vaccine is initiated after 2035. If administering two different HPV vaccines to the same individual is acceptable, the optimal strategy is the mixed-vaccine strategy of one-dose 2vHPV and one-dose 9v HPV vaccine.

**Implications of all the available evidence**

Our study supports immediate vaccination with 2vHPV vaccine followed by switching to the 9vHPV vaccine, if the current regulations on administering the same vaccine for both doses need to be followed. Adopting a mixed vaccine schedules involving one immediate dose of 2vHPV vaccine and one delayed dose of 9vHPV vaccine is a promising option to expand both the health and economic benefits in the context of constrained 9vHPV vaccine supply. These findings may also provide policy evidence for other countries that have not yet introduce 9v HPV vaccines.

## Introduction

Globally, cervical cancer is the fourth most common cancer in females with an estimated 662,000 new cases and 348,000 deaths in 2022.^1^ China has the largest burden of cervical cancer cases in the world, with 23% of global cases in 2022.^2^ Cervical cancer incidence in China increased by 7.3% per year during 2000–2018.^2^ Human papillomavirus (HPV) vaccination is a highly effective long-term intervention for preventing cervical cancer and a foundational pillar of the World Health Organisation (WHO) global strategy to accelerate the elimination of cervical cancer. As of December 2023, 141 (73%) of the 194 WHO member states had introduced HPV vaccination into their national immunisation programmes (NIP), however, China has yet to do so. In January 2023, China issued an action plan to accelerate cervical cancer elimination (2023–2030),^3^ which proposed to promote pilot HPV vaccination programs for school-age girls and pledged to accelerate the approval for new HPV vaccines.

China has approved five brands of HPV vaccines, including three bivalent (2vHPV, Cervarix®, Cecolin®, and Walrinvax™) vaccines, a quadrivalent (4vHPV, Gardasil®) vaccine, and a nonavalent (9vHPV, Gardasil®9) vaccine. The 2vHPV vaccines offer protection against HPV 16 and 18, which are the most common causes of cervical cancer. With multiple manufacturers producing the 2vHPV vaccines, their production capacity is sufficient for current national demand for routine vaccination of girls in China. Moreover, competition among manufacturers may drive down prices of 2vHPV vaccines in compliance with the requirements of the NIP. Some pilot sites in China have already introduced free 2vHPV vaccination programmes for adolescent girls.^4^ In contrast, the 9vHPV vaccine provides protection against additional HPV types 6, 11, 31, 33, 45, 52, and 58. However, with only one 9vHPV vaccine in the market, its limited supply and higher cost pose significant challenges for its widespread use in the Chinese NIP. Currently, at least four novel 9vHPV vaccine candidates are undergoing phase III clinical trials. When these trials are completed, their eventual market introduction is expected to alleviate the current vaccine supply pressure.

Some high-income countries, including the USA, Canada, Austria, and Italy, have replaced 2vHPV or 4vHPV vaccines with the 9vHPV vaccine in their NIP,^5^ as studies have established the superior efficacy and cost-effectiveness of the 9vHPV vaccine in these countries.^6^, ^7^, ^8^, ^9^ Although three studies have evaluated the cost-effectiveness of switching from 2vHPV to 9vHPV vaccines in China,^10^, ^11^, ^12^ they are all based on the previous three-dose vaccination schedule rather than the currently recommended two- or one-dose vaccination schedule for adolescent girls. Moreover, some Chinese women have been hesitant to receive HPV vaccination, preferring to wait for the 9vHPV vaccine.^13^ Considering the current high price and limited supply of 9vHPV vaccines in China, studies are needed to inform the decision between immediately initiating widespread 2vHPV vaccination and waiting for cheaper 9vHPV vaccines to become widely available, which is not addressed by any existing study. Additionally, Quebec (Canada) has adopted a mixed schedule of using both 2vHPV and 9vHPV vaccines from 2018, which could spare doses of the supply-limited and expensive 9vHPV vaccines while still providing most of the benefits.^14^ However, no studies have assessed the potential cost-effectiveness of mixed vaccine schedules in China.

To address these knowledge gaps, our study focuses on assessing population-level effectiveness and cost-effectiveness of switching from 2vHPV to 9vHPV vaccination in China, and identifying the optimal vaccination strategy, taking into account the year when 9vHPV vaccine becomes available in sufficient quantities to be included in the NIP, the level of cervical screening, and the potential inclusion of mixed vaccination schedules.

## Methods

We used a hybrid model consisting of a dynamic HPV transmission model and a static natural history model to estimate the health outcomes and costs of different vaccination strategies. The details of the model have been presented previously^15^ and are summarised in the Appendix (pp 2-3 and Figure S1). Briefly, the deterministic age-structured compartmental dynamic model was built to simulate HPV transmission between males and females and to project the number of HPV infections. The natural history model was then used to simulate the natural history of cervical cancer and its precursors to estimate the number of cervical cancer cases and deaths. The model has been previously calibrated using Chinese epidemiological data on high-risk HPV type prevalence, cervical cancer incidence and mortality in 2015, and HPV type distribution in women with normal cervical cytology, low-grade cervical precancerous lesions, high-grade cervical precancerous lesions, and invasive cervical cancer.^15^ We considered all women living or projected to be born in China during 2023–2100. The population was stratified by area of residence (urban and rural), sex, sexual activity (high, low, none), and age (single year of age from 0 to 84 years, and ≥85 group). All individuals were simulated until the end of their lives. Model assumptions and results were reported following the HPV-FRAME checklist^16^ and the CHEERS checklist.^17^

### Alternative strategies

Several 9vHPV vaccine candidates are in Phase III clinical trials, expected to be completed in 2027–2029 at the earliest.^18^^,^^19^ Therefore, we assumed that sufficient quantities of the 9vHPV vaccine would become available for the NIP between 2030 and 2050, depending on the development and production schedule. Under different year when 9vHPV vaccine becomes available (2030, 2035, 2040, 2045, and 2050, referred to as the 9vHPV initiation year), we evaluated five same-vaccine strategies and two mixed-vaccine strategies: (1) no vaccination; (2) immediate routine 2vHPV vaccination from 2023; (3) routine 9vHPV vaccination from the 9vHPV initiation year; (4) immediate routine 2vHPV vaccination from 2023 with a switch to 9vHPV vaccine in the 9vHPV initiation year; (5) routine 9vHPV vaccination from the 9vHPV initiation year with multi-age cohort catch-up vaccination delivered in a single year; (6) mixed two-dose schedule (first dose of 2vHPV vaccine targeting girls aged 12 and a second dose of 9vHPV vaccine in the 9vHPV initiation year) from 2023, and switching to two-dose routine 9vHPV vaccination in the 9vHPV initiation year; and (7) adding the restriction of a 5-year maximum interval between two doses based on the strategy 6 (i.e., if 9vHPV vaccine is not available 5 years after a birth cohort received the first dose of 2vHPV vaccine, the cohort will instead receive a second dose of 2vHPV vaccine in that year). Routine HPV vaccination targeted girls aged 12 years with a two-dose schedule at 90% coverage. Catch-up vaccination targeted girls aged 13–18 years at 90% coverage, with a two-dose schedule for girls aged 13–14 years and a three-dose schedule for girls aged ≥15 years. The details of these vaccination strategies are described in the Appendix (Figures S2 and S3).

We evaluated the vaccination strategies under two different screening scenarios. The first scenario (status quo screening) is the current programme with 3-yearly cytology-based screening at coverage of 26.6% in urban areas and 19.3% in rural areas (Figure S4).^20^ The second scenario (improved HPV-based screening) assumed a switch to 5-yearly HPV-based screening in 2022, with linearly increasing age-specific uptake for the target population of women aged 35–64 years from the status quo in 2021, to 70% in 2030, followed by a 1% increase every year till 90% is reached. This improved scenario was based on the target of reaching 70% screening coverage in women aged 35–64 years by 2030 in China's action plan^3^ and the recommendation of HPV testing as the primary method for cervical cancer screening by WHO^21^ and the Chinese Preventive Medicine Association.^22^ The screening coverage was assumed to continue to increase after the target is reached, until reaching 90%, because five-year screening coverage in some countries has already surpassed 70%, whereas very few countries have achieved screening coverage exceeding 90%.^23^^,^^24^

### Inputs and assumptions

A full-course vaccine schedule was assumed to provide 100% protection against vaccine-targeted HPV types. One-dose vaccine was conservatively assumed to provide 85% protection against vaccine-targeted HPV types based on the lower bound efficacy observed after 10 years of one-dose HPV vaccine.^25^ Therefore, in mixed-vaccine two-dose strategies of 2vHPV and 9vHPV, the first dose of 2vHPV was assumed to provide 85% protection against HPVs 16 and 18; the second dose of 9vHPV in the mixed schedule was assumed to provide 100% protection against HPVs 16 and 18, and 85% protection against the seven genotypes included in 9vHPV but omitted in 2vHPV. Vaccine-induced immunity was assumed to be lifelong.

Model parameters are listed in the Appendix Table S1. All unit costs were converted to 2022 US dollars, adjusting for inflation using China's Consumer Price Index for health care and currency conversion using the average US dollar to Chinese Yuan exchange rate in 2022 (1.00 US dollar = 6.70 Chinese Yuan). Current market prices for 2vHPV and 9vHPV vaccines are $49.10 and $193.73 per dose, respectively. We assumed lower national tender prices ($4.32 and $17.05 per dose for 2vHPV and 9vHPV vaccines) based on prices paid by the Pan American Health Organisation (PAHO) Revolving Fund,^26^ given China's comparable income level to many PAHO countries^27^ and large market size. We assumed vaccine delivery costs to be $4.02 per dose.^28^ The treatment costs for cervical intraepithelial neoplasia (CIN) and cervical cancer were collected in our nationwide multicenter cross-sectional, hospital-based survey.^29^ Detailed cost assumptions are available in the Appendix (p3).

### Main analysis

The impact and cost-effectiveness of different vaccination strategies were estimated under varied screening scenarios and 9vHPV initiation years. For population-level impact, we estimated cervical cancer cases and deaths averted by each vaccination strategy compared with no vaccination. We also estimated age-standardised annual cervical incidence during 2023–2100 using Segi's world standard population and identified the year of cervical cancer elimination, defined as the first year when the incidence falls below 4/100,000 women.^30^ Considering that the 9vHPV vaccine will only become available to some cohorts at an older age, we further estimated the effect of age at receiving the second dose of 9vHPV vaccine on cervical cancer cases, deaths, and costs. Specifically, we evaluated the outcomes of administering a first dose of the 2vHPV vaccine at age 12, followed by a second dose of the 9vHPV vaccine at various ages, including 12, 15, 20, 25, 30, 35, and 40 years. For the cost-effectiveness analysis, we estimated the incremental costs and effectiveness of each vaccination strategy compared with no vaccination, as well as those of switching from 2vHPV to 9vHPV vaccination compared with maintaining 2vHPV vaccination. The effectiveness was quantified using quality-adjusted life-years (QALYs), taking into account health state utility weights. Incremental cost-effectiveness ratios (ICERs) were calculated as incremental cost per additional QALY gained of a strategy compared to the next most costly non-dominated strategy, to identify the optimal strategy. We performed the analysis using a societal perspective. The time horizon of the analysis was 2023–2100, and all cohorts were followed up until the end of their lives. Both costs and QALYs were discounted to 2022 at an annual rate of 3%.^31^ The previous cost-effectiveness threshold of 1–3 times gross domestic product (GDP) per capita suggested at one time by WHO's Commission on Macroeconomics and Health is now considered to be high for low- and middle-income countries (LMICs).^32^^,^^33^ Following a recent global analysis,^34^ we applied the 0.51 times GDP per capita of China in 2022 (0.51∗$12,791 = $6523)^35^ as the cost-effectiveness threshold. All analyses were performed in R (version 4.2.0).

### Sensitivity analysis

In our sensitivity analysis, we conservatively assumed 40% vaccination coverage for the second dose in mixed-vaccine strategies, reflecting potential lower complete vaccination rates when extending vaccination intervals. This assumption was based on the lowest coverage observed (39% in Costa Rica) for the second dose among LMICs with HPV vaccination programmes that reached 90% coverage for the first dose in 2019.^36^ We also used current vaccine prices in cost-effectiveness analysis to assess the effect of varying vaccine prices. Additionally, we adopted an alternative discount rate recommended by WHO^31^ using 3% and 0% discounting for costs and QALYs, respectively. For the elimination year, we also calculated the age-standardised incidence using the World Female Population 2015 (ages 0–99 years), which is recommended for comparisons between countries and for incidence calculations used to inform WHO strategic planning for cervical cancer elimination.^37^ In one-way deterministic sensitivity analyses, we varied key parameters in the model over their plausible ranges to quantify the impact of uncertainty in individual input parameters on the results (Table S1). Probabilistic sensitivity analysis was conducted by performing 1000 Monte Carlo simulations to sample parameter values from their respective distributions (Table S1), including screening sensitivity, precancerous lesions management, treatment efficacy, costs, and utilities assumptions, and to estimate outcomes. The results of the probabilistic sensitivity analysis were used to calculate the 10th and 90th percentiles (80% uncertainty interval) of model results.

### Role of the funding source

The funder of the study had no role in study design, data collection, data analysis, data interpretation, or writing of the report.

## Results

### Same-vaccine strategies

Under base-case assumptions, compared with maintaining 2vHPV vaccination, switching to 9vHPV was cost-saving irrespective of screening scenarios and 9vHPV initiation years (Table 1, status quo screening: net cost saving $2589–5211 million; improved HPV-based screening: net cost saving $1852–3789 million). Compared to no vaccination, routine 2vHPV vaccination from 2023 would reduce cervical cancer incidence in 2100 by 56.49% (80% UI = 56.28%–56.71%) under status quo screening and 66.20% (80% UI = 65.07%–67.19%) under improved HPV-based screening scenario, respectively (Table 1). Switching to 9vHPV vaccination would further reduce cervical cancer incidence, especially if 9vHPV vaccination is initiated earlier. Specifically, switching from 2vHPV to 9vHPV vaccination is estimated to reduce cervical cancer incidence in 2100 by 78.49%–84.45% under status quo screening and 84.05%–87.69% under improved HPV-based screening scenario compared to no vaccination depending on the 9vHPV initiation year (Table 1).

**Table 1:** Effectiveness and cost-effectiveness of switching from 2vHPV to 9vHPV vaccination by the 9vHPV initiation year.

9vHPV initiation year	Vaccination strategies	Incidence in 2100 (/10^5^ women)	Compared with no vaccination			Compared with maintaining 2vHPV		
			Reduction in incidence in 2100 (%)	Cases averted (million)	Deaths averted (million)	Reduction in incidence in 2100 (%)	Cost saving ($ million)	QALYs gained (million)
**Status quo screening**								
–	Maintaining 2vHPV	10.23 (9.68–10.70)	56.49 (56.28–56.71)	6.82 (6.50–7.08)	2.38 (2.30–2.48)	–	–	–
2030	2vHPV to 9vHPV	3.66 (3.46–3.83)	84.45 (84.36–84.55)	10.35 (9.84–10.77)	3.78 (3.63–3.93)	64.26 (64.18–64.34)	5211 (4034 to 6460)	1.57 (1.51–1.64)
2035	2vHPV to 9vHPV	3.76 (3.55–3.93)	84.03 (83.94–84.13)	10.09 (9.60–10.50)	3.67 (3.53–3.82)	63.29 (63.22–63.37)	4335 (3374 to 5371)	1.30 (1.25–1.36)
2040	2vHPV to 9vHPV	3.97 (3.76–4.16)	83.11 (83.02–83.21)	9.85 (9.37–10.25)	3.58 (3.44–3.72)	61.18 (61.11–61.25)	3639 (2839 to 4507)	1.09 (1.05–1.14)
2045	2vHPV to 9vHPV	4.39 (4.15–4.60)	81.32 (81.22–81.44)	9.63 (9.16–10.02)	3.49 (3.35–3.63)	57.07 (57.01–57.14)	3074 (2405 to 3804)	0.92 (0.88–0.96)
2050	2vHPV to 9vHPV	5.06 (4.79–5.29)	78.49 (78.37–78.62)	9.40 (8.95–9.79)	3.40 (3.27–3.53)	50.57 (50.48–50.67)	2589 (2030 to 3200)	0.77 (0.74–0.80)
**Improved HPV-based screening**								
–	Maintaining 2vHPV	3.11 (2.68–3.68)	66.20 (65.07–67.19)	3.17 (2.87–3.55)	0.98 (0.90–1.08)	–	–	–
2030	2vHPV to 9vHPV	1.13 (0.97–1.34)	87.69 (87.24–88.09)	4.25 (3.81–4.80)	1.36 (1.25–1.52)	63.59 (63.23–63.95)	3789 (2744 to 4864)	0.48 (0.40–0.62)
2035	2vHPV to 9vHPV	1.17 (1.01–1.39)	87.23 (86.80–87.60)	4.17 (3.74–4.71)	1.34 (1.23–1.49)	62.21 (61.87–62.61)	3119 (2271 to 3986)	0.40 (0.33–0.51)
2040	2vHPV to 9vHPV	1.25 (1.09–1.47)	86.37 (85.98–86.71)	4.10 (3.68–4.63)	1.31 (1.20–1.46)	59.68 (59.27–60.21)	2601 (1898 to 3322)	0.34 (0.28–0.43)
2045	2vHPV to 9vHPV	1.37 (1.19–1.60)	85.13 (84.78–85.46)	4.03 (3.62–4.55)	1.28 (1.18–1.43)	56.02 (55.42–56.73)	2195 (1607 to 2798)	0.28 (0.23–0.36)
2050	2vHPV to 9vHPV	1.47 (1.27–1.72)	84.05 (83.63–84.43)	3.96 (3.56–4.47)	1.26 (1.16–1.40)	52.81 (52.19–53.45)	1852 (1361 to 2356)	0.24 (0.20–0.30)

The numbers of cervical cancer cases and deaths averted, cost saving and QALYs gained are estimated over the lifetime of people who lived in 2023–2100. The values in the parentheses represent the 80% uncertainty intervals. The improved HPV-based screening scenario represents switching to 5-yearly HPV-based screening in 2022, with linearly increasing age-specific uptake from status quo in 2021 to 70% in 2030, followed by a 1% increase every year till 90% is reached.

HPV, Human Papillomavirus; 2vHPV, bivalent HPV vaccine; 9vHPV, nonavalent HPV vaccine; QALY, quality-adjusted life-year.

**Fig. 1:**
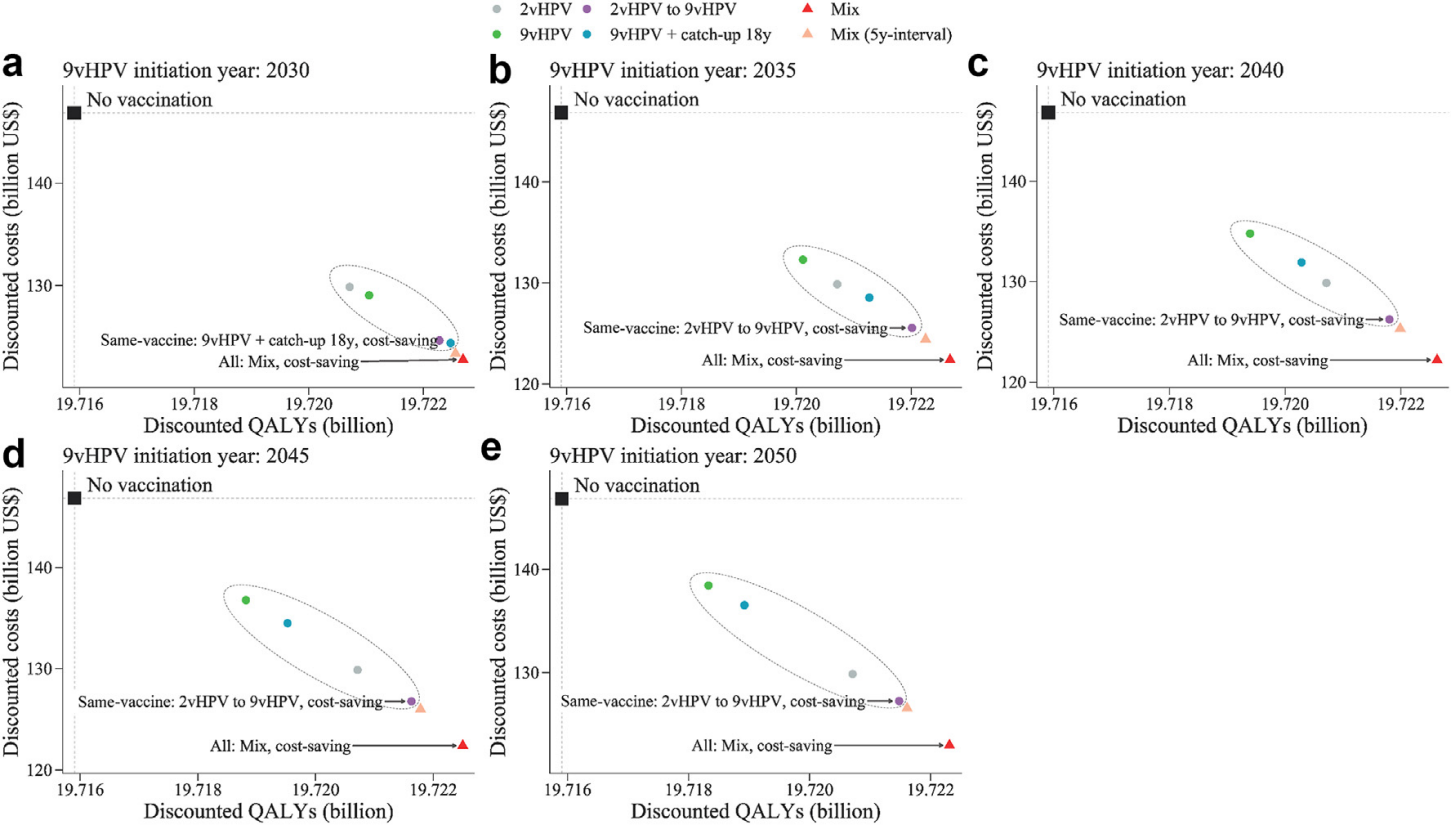
**Cost-effectiveness for different vaccination strategies compared with no vaccination under status quo screening, with 3% discounting. The 9vHPV initiation year was (a) 2030, (b) 2035, (c) 2040, (d) 2045, and (e) 2050**. The black squares indicate the reference strategy of no vaccination. The circles inside dashed ovals indicate the same-vaccine strategies, while the triangles indicate the mixed-vaccine strategies. The “mix” strategy represents mixed two-dose schedule (first dose of 2vHPV vaccine targeting girls aged 12 and a second dose of 9vHPV vaccine in the 9vHPV initiation year) from 2023, and switching to two-dose routine 9vHPV vaccination when 9vHPV vaccine is available for national vaccination. The “mix (5y-interval)” represents the strategy that adds the restriction of a 5-year maximum interval between two doses based on the “mix” strategy. The strategies in the upper left quadrant are dominated by the strategies in the lower right quadrant. The labels represent the dominant vaccination strategy in the same-vaccine strategies and all strategies (including same-vaccine and mixed-vaccine strategies), respectively. Because the dominant strategies are cost-saving, the ICERs are negative and not shown. HPV, Human Papillomavirus; 2vHPV, bivalent HPV vaccine; 9vHPV, nonavalent HPV vaccine; QALY, quality-adjusted life-year.

**Fig. 2:**
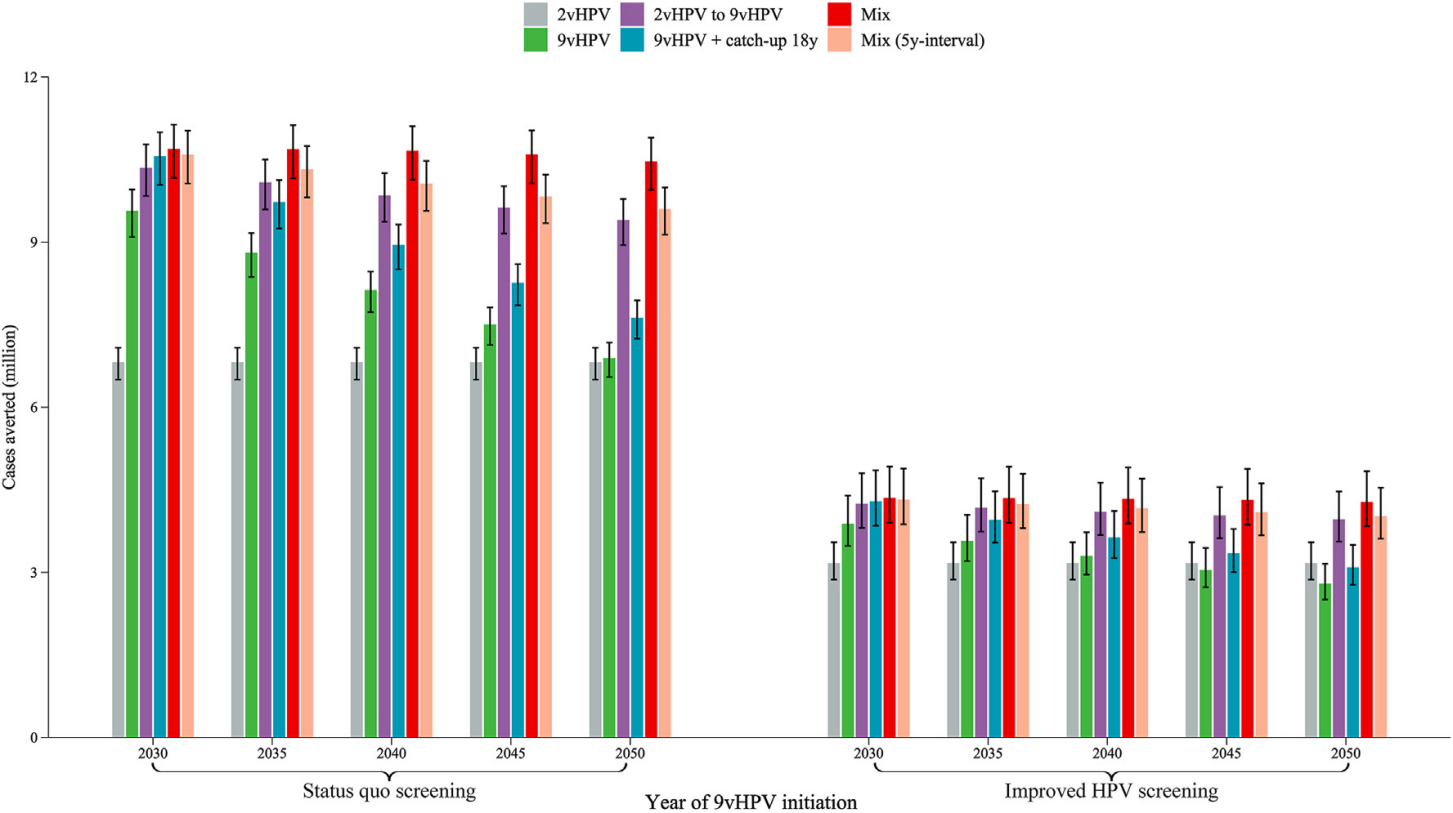
**Cervical cancer cases averted by different vaccination strategies compared with no vaccination under two screening settings**. Error bars represent the 80% uncertainty intervals of cases averted compared with no vaccination. The “mix” strategy represents mixed two-dose schedule (first dose of 2vHPV vaccine targeting girls aged 12 and a second dose of 9vHPV vaccine in the 9vHPV initiation year) from 2023, and switching to two-dose routine 9vHPV vaccination when 9vHPV vaccine is available for national vaccination. The “mix (5y-interval)” represents the strategy that adds the restriction of a 5-year maximum interval between two doses based on the “mix” strategy. The improved HPV-based screening scenario represents switching to 5-yearly HPV-based screening in 2022, with linearly increasing age-specific uptake from status quo in 2021 to 70% in 2030, followed by a 1% increase every year till 90% is reached. HPV, Human Papillomavirus; 2vHPV, bivalent HPV vaccine; 9vHPV, nonavalent HPV vaccine.

The 9vHPV initiation year would affect the optimal vaccination strategy. In same-vaccine strategies, the optimal strategy was either “switching from 2vHPV to 9vHPV” or “9vHPV vaccination with a catch-up”, both of which required initiating 9vHPV as soon as the vaccine became available. When assuming the initiation of the 9vHPV vaccine occurs in 2030, the optimal strategy was vaccinating using 9vHPV with a catch-up to age 18, which was cost-saving and dominated all other same-vaccine strategies (Fig. 1 and Figure S5). This strategy would result in slightly higher health and economic benefits than switching from 2vHPV to 9vHPV vaccination, and substantially higher benefits than maintaining 2vHPV vaccination or vaccinating 9vHPV without a catch-up. However, as the 9vHPV vaccine was initiated later, the decline in the benefits of 9vHPV vaccination regardless of a catch-up, was substantially greater than that of switching from 2vHPV to 9vHPV. Consequently, when assuming the initiation of the 9vHPV vaccine occurs after 2035, the optimal strategy was immediate 2vHPV vaccination with a later switch to 9vHPV (Fig. 1 and Figure S5). Compared with maintaining 2vHPV vaccination, the above optimal strategies would avert 0.79–3.74 million additional cervical cancer cases and 0.28–1.48 million deaths, save $1852–5460 million net costs, and gain 0.24–1.76 million QALYs over the lifetime of people who lived in 2023–2100, depending on the screening scenario and the 9vHPV initiation year (Fig. 2, Figure S6 and Tables S2 and S3). Additionally, under status quo screening, the optimal strategies would achieve cervical cancer elimination by 2081, 2090, and 2099 when the 9vHPV initiation year was 2030, 2035, and 2040, respectively, but could not achieve elimination when the 9vHPV initiation year was after 2045. Under improved HPV-based screening, the optimal strategies would achieve cervical cancer elimination by 2062–2066 (Table 2).

**Table 2:** Estimated cervical cancer elimination year of different vaccination strategies by the 9vHPV initiation year.

9vHPV initiation year	vaccination strategies					
	Maintaining 2vHPV	Waiting for 9vHPV	2vHPV to 9vHPV	9vHPV + catch-up	Mixed-vaccine strategy (no interval restriction)	Mixed-vaccine strategy (5y-interval)
**Status quo screening**						
2030	–	2090 (2088–2091)	2085 (2083–2086)	2081 (2080–2083)	2081 (2079–2082)	2082 (2080–2083)
2035	–	2097 (2095–2100)	2090 (2088–2093)	2088 (2086–2090)	2081 (2079–2082)	2085 (2084–2087)
2040	–	–	2099 (2095–2100)	2096 (2093–2100)	2081 (2079–2082)	2091 (2089–2095)
2045	–	–	–	–	2081 (2080–2082)	–
2050	–	–	–	–	2082 (2081–2084)	–
**Improved HPV-based screening**						
2030	2066 (2061–2073)	2068 (2064–2068)	2061 (2059–2065)	2062 (2058–2062)	2060 (2057–2061)	2061 (2058–2063)
2035	2066 (2061–2073)	2072 (2069–2073)	2062 (2060–2066)	2067 (2063–2068)	2060 (2057–2061)	2061 (2059–2065)
2040	2066 (2061–2073)	2077 (2073–2078)	2063 (2061–2068)	2071 (2068–2073)	2061 (2057–2061)	2062 (2060–2066)
2045	2066 (2061–2073)	2082 (2078–2083)	2065 (2061–2070)	2076 (2073–2077)	2061 (2058–2062)	2063 (2061–2068)
2050	2066 (2061–2073)	2087 (2084–2088)	2066 (2061–2071)	2081 (2077–2082)	2061 (2060–2064)	2065 (2061–2070)

“–” indicates that cervical cancer will not be eliminated by 2100 by adopting this strategy. The values in the parentheses represent the 80% uncertainty intervals of the elimination years. The improved HPV-based screening scenario represents switching to 5-yearly HPV-based screening in 2022, with linearly increasing age-specific uptake from status quo in 2021 to 70% in 2030, followed by a 1% increase every year till 90% is reached.

HPV, Human Papillomavirus; 2vHPV, bivalent HPV vaccine; 9vHPV, nonavalent HPV vaccine.

### Mixed-vaccine strategies

Adopting mixed-vaccine strategies with one dose of 2vHPV first and one dose of 9vHPV when 9vHPV vaccines become available in sufficient quantities for national vaccination would cost less and gain more QALYs than all same-vaccine strategies irrespective of the screening scenarios and the 9vHPV initiation year (Fig. 1 and Figure S5). Importantly, the effectiveness of this mixed-vaccine strategy in eliminating cervical cancer was minimally affected by the year of 9vHPV initiation. The strategy would achieve elimination by 2081–2082 (Table 2, 0–18 years earlier compared with the optimal strategies in same-vaccine strategies) under status quo screening and by 2060–2061 (2–5 years earlier) under improved screening, depending on the 9vHPV initiation year. Additionally, health and economic benefits of the mixed-vaccine strategy would decrease with increasing vaccination age at the second dose. However, even when the second dose of the 9-valent vaccine is administered up to the age of 40 years, the mixed-vaccine strategy would still avert more cases and reduce incremental costs related to cervical cancer compared with two-dose routine 2vHPV vaccination (Figure S7).

If the maximum interval between two doses was restricted to 5 years, the additional health and economic benefit of a mixed-vaccine strategy compared to a same-vaccine strategy would be reduced. Compared with the optimal strategies in same-vaccine strategies, this mixed-vaccine strategy would accelerate elimination by 1–2 years under status quo screening and by 1–5 years under improved screening, respectively (Table 2). These benefits were reduced when the interval between two doses was restricted, primarily because some women would change from receiving one-dose 2vHPV and one-dose 9vHPV vaccine to receiving two doses of the 2vHPV vaccine. Fewer vaccinated women would receive one-dose 2vHPV and one-dose 9vHPV vaccine in the mixed-vaccine strategy with 5-year maximum interval between doses compared to without restricted intervals (5.8–7.8% vs. 11.0–36.5%, Figure S8).

### Sensitivity analysis

Even if vaccination coverage of the second dose for mixed-vaccine strategies is reduced to 40%, the mixed-vaccine strategy without restrictions on the length of intervals between doses remained the optimal strategy (Figures S9–S10). When assuming current market vaccine prices, switching to 9vHPV vaccination or mixed-vaccine strategies was not cost-effective, thus the optimal strategy was maintaining 2vHPV vaccination (Figures S11–S12). The optimal strategies when considering either same-vaccine strategies or all strategies did not change when using 3% and 0% discounting for costs and QALYs in the cost-effectiveness analysis (Figures S13–S14). The elimination year calculated using World Female Population 2015 was shown in Table S4. Deterministic sensitivity analysis showed that the model was sensitive to treatment costs, utilities, screening sensitivity, vaccination coverage, and maximum achievable screening coverage (Figures S15–S18). However, switching from 2vHPV to 9vHPV vaccination always remained cost-saving compared with maintaining 2vHPV vaccination. In the probabilistic sensitivity analysis, the uncertainty ranges around the cases averted, deaths averted and the elimination year were small. Switching from 2vHPV to 9vHPV vaccination remained more effective and less costly than maintaining 2vHPV vaccination (Figures S19–S20). Similarly, the mixed-vaccine strategy remained more effective and less costly than switching from 2vHPV to 9vHPV vaccination (Figures S21–S22).

## Discussion

In this transmission-dynamic modeling study, we found that switching to 9vHPV vaccination was cost-saving compared to maintaining 2vHPV vaccination in China, and determined the optimal vaccination strategy prior to 9vHPV vaccines becoming available for national vaccination. In the same-vaccine strategies, the optimal vaccination strategy is to wait for routine 9vHPV vaccination with a catch-up to age 18 if 9vHPV vaccine is initiated in 2030 and to vaccinate using 2vHPV immediately with a switch to 9vHPV if 9vHPV vaccine is initiated after 2035. Adopting this strategy would result in more than $1852 million cost savings and 237,000 QALYs gained over the lifetime of people who lived in 2023–2100 compared to maintaining 2vHPV vaccination. If administering two different HPV vaccines to the same individual is acceptable, the optimal strategy would become the mixed-vaccine strategy of one-dose 2vHPV and one-dose 9vHPV vaccine. This mixed-vaccine strategy would further represent a saving of more than $1336 million costs and 87,000 QALYs gained compared to the above optimal strategy in the same-vaccine strategies.

Our study demonstrated that switching from 2vHPV to 9vHPV vaccination was a cost-saving strategy compared to maintaining 2vHPV vaccination under the assumption of negotiated vaccine prices. These results were robust under both status quo screening and HPV-based screening with improved coverage. Our findings were broadly in line with published studies in other countries. In Italy, the switch to 9vHPV vaccination of school-aged girls was cost-saving compared with continuing 2vHPV vaccination at the public sector price.^6^ Similarly, studies in some LMICs, such as South Africa,^38^ India,^39^ and Thailand,^40^ have found 9vHPV vaccination was cost-effective compared to the 2vHPV vaccination. Most cost-effectiveness studies indicated that 9vHPV vaccination required a reduction from market prices to be selected for NIP, and the incremental price difference between the 9vHPV and 2vHPV vaccines given our negotiated price assumptions fell within the range of acceptable additional cost in these studies. For instance, replacing 2vHPV with 9vHPV vaccine was estimated to be cost-effective given the additional costs per dose were less than $19 in Norway^41^ and $30 in Singapore,^42^ respectively. Notably, switching to 9vHPV vaccination was not cost-effective at the current high commercial price ($193.73 per dose) according to our sensitivity analysis. The price of the 2vHPV vaccine purchased by the Guangdong provincial government has been reduced to $17.3 per dose through provincial public tendering and centralised procurement,^43^ and is likely to further be reduced through national centralised procurement in the future. In contrast, the 9vHPV vaccine is predominantly purchased privately at considerably higher prices, which is a substantial obstacle for the switch to national 9vHPV vaccination in China. In 2023, China's National Medical Products Administration released technical guidelines for clinical trials of HPV vaccines.^44^ These guidelines simplify the trial design for high-valency iterative vaccines, i.e., those developed based on the platforms of previous-generation vaccines, to facilitate HPV vaccine development and improve the efficiency of technical review. This policy provides opportunities for vaccine manufacturers to expedite the marketing of new 9vHPV vaccines and to improve vaccine accessibility and affordability in China.

Our model projections support the prompt administration of currently available vaccines rather than waiting for 9vHPV to become available. In our study, immediate routine 2vHPV vaccination with a subsequent switch to 9vHPV could cost less and gain more QALYs than waiting for routine 9vHPV vaccination, due to the protection provided by 2vHPV vaccines to some girls who do not currently have access to 9vHPV vaccines. The readily available 2vHPV vaccines already protect against HPV types 16 and 18, which account for up to 84.5% of cervical cancer cases in China.^45^ This indicates that achieving high 2vHPV vaccine coverage among young girls should be given priority to provide them with adequate and timely protection against cervical cancer. Additionally, adding catch-up vaccination in the 9vHPV vaccine initiation year could produce health and economic benefits compared to routine 9vHPV vaccination. These benefits of catch-up 9vHPV vaccination to age 18 will offset the losses in waiting for routine 9vHPV vaccination if 9vHPV vaccine became available in 2030, but cannot compensate for the losses once 9vHPV vaccine became available after 2035. This is mainly because the risks of HPV infection and missing catch-up vaccination during the longer waiting period will exceed the benefits of enhanced protection against a broader spectrum of HPV types.

Our study also highlights the value of mixed-vaccine strategies of one dose of 2vHPV now and one dose of 9vHPV when the 9vHPV vaccine becomes widely accessible, which could avert more cervical cancer cases than all same-vaccine strategies. One potential reason is that the mixed-vaccine strategy combines the advantages of vaccinating adolescent girls with a single dose to offer early protection against the most carcinogenic types (HPV 16 and 18), and administering 9vHPV vaccines with the second dose to provide additional protection against the other 7 HPV types. Several clinical trials have shown that mixed schedules combining different vaccines are safe and highly immunogenic.^46^, ^47^, ^48^, ^49^, ^50^ For example, Gilca et al. in Canada^49^ found that a mixed schedule of 2vHPV and 9vHPV has an acceptable safety profile. Although the mixed schedule induces lower antibody titres against the 7 HPV types not included in 2vHPV compared to two doses of 9vHPV, the titres are still sufficiently high to be protective against all 9 HPV types included in 9vHPV.^49^ Based on these results, Quebec, Canada has implemented a mixed vaccination schedule from 2018.^14^ Furthermore, although no clinical trials have reported on the efficacy of mixed vaccination, single-dose 9vHPV vaccination included in mixed schedules provided 95.5% efficacy against all 9 HPV types for at least 3 years,^51^ suggesting mixed schedules may provide durable protection. Consequently, for countries where 9vHPV vaccination has not been available for national vaccination, like China, the mixed-vaccine strategy may be a flexible choice with optimal protection at a better cost. This strategy may also reduce vaccine hesitancy towards 2vHPV vaccines due to preference for 9vHPV vaccine, as well as mitigate health inequalities among different birth cohorts who may receive different HPV vaccines.

However, given the uncertainty around when 9vHPV vaccines will become available in sufficient quantities for national vaccination, females receiving the mixed schedule may have to wait a long time for the second dose. The long interval may raise concerns about infections occurring during the delay between doses, as well as declines in vaccination coverage of the second dose. Nevertheless, our sensitivity analysis showed that even with pessimistic assumptions of 85% single-dose vaccine efficacy and 40% coverage of the second dose, the mixed-vaccine strategy remained cost-saving compared with all same-vaccine strategies. Post-hoc analyses of participants in the Costa Rica Vaccine Trial (CVT) showed sustained high efficacy of one-dose 2vHPV vaccine for up to 11 years.^52^ This suggests that even with long intervals between doses, the mixed schedule will likely provide long-lasting protection against HPV types 16 and 18. WHO has proposed that there is no recommended maximum interval between doses of the HPV vaccine,^53^ and has suggested that an extended interval of 3–5 years could be considered to alleviate vaccine supply shortages.^54^ Considering that longer intervals between doses may present more programmatic challenges in acceptability and implementation, our study also includes a mixed-vaccine strategy restricting the maximum interval to 5 years. This strategy still prevented more cervical cancer cases than same-vaccine strategies, even though some females would receive a second dose of 2vHPV vaccine instead of 9vHPV because the 9vHPV vaccine is assumed not to become available in time.

There are several strengths to our study. First, our study used a transmission dynamic model to assess the vaccination strategies, so we were able to capture herd effects, which are important when assessing catch-up vaccination. Second, we considered several future 9vHPV vaccine availability scenarios and screening settings, which are grounded in current vaccine development schedules and timelines of clinical trials,^18^^,^^19^^,^^55^ as well as current status and long-time targets for the national screening programme. Thus, our strategies are likely to be feasible in the Chinese context. Third, to our knowledge, this is the first cost-effectiveness analysis including scenarios of mixed 2vHPV and 9vHPV vaccination strategies. Our study could thus inform flexible vaccine use in response to vaccine availability constraints.

Our study also has several limitations. First, the model did not incorporate the potential benefits of vaccination against genital warts and non-cervical cancers such as anal cancer, vaginal cancer, and head and neck cancer due to lack of data. This may lead to conservative estimates of the health benefits and cost-effectiveness of HPV vaccines, particularly 9vHPV vaccines. However, this conservative estimate has already indicated that switching from bivalent to 9vHPV vaccination is cost-saving. Second, we did not include the scenarios of male vaccination since this is not a current priority for policy-makers in China given supply limitations. According to WHO's global strategy for cervical cancer elimination,^30^ the primary target is to vaccinate girls who could benefit directly from HPV vaccines given the current constrained vaccine supply. If the vaccine shortage is alleviated in the future, vaccination of boys may be considered in China, which could expand the health benefits in preventing male HPV-related diseases as well as enhance cervical cancer protection for women through reducing HPV transmission. Third, the evidence for efficacy of mixed-vaccine strategies is still sparse, so we instead assumed efficacy based on the findings of one-dose vaccines. However, our sensitivity analysis shows that varying the efficacy has a small impact on the results of cost-effectiveness analysis.

### Conclusion

The switch to 9vHPV vaccination is likely cost-saving compared with maintaining 2vHPV vaccination once sufficient doses become available. Such a switch is likely to have a significant impact on reducing the cervical cancer burden in China. Our study supports immediate vaccination with the 2vHPV vaccine followed by switching to the 9vHPV vaccine if the current regulations on administering the same vaccine need to be followed. Adopting mixed vaccination schedules involving one immediate dose of 2vHPV and one delayed dose of 9vHPV vaccine is a promising option to expand both the health and economic benefits in the context of constrained 9vHPV vaccine supply.

## Contributors

FZ, CW, and MG conceived and designed the study. FZ and MJ contributed to funding acquisition of the study. MG and TY accessed and verified all reported data. MG, SH, XZ, TY, and YH contributed to the analysis and visualization of the study. MG drafted the manuscript. MJ, YL, and YQ critically revised the manuscript for intellectual content. All authors approved the final version of the manuscript. All authors had full access to all the data in the study and had final responsibility for the decision to submit for publication.

## Data sharing statement

This study does not involve any patient data or participant data. Readers can access the data used in this study from the links to public domain resources provided in the Methods. The code used to generate the reported estimates is sensitive, interested parties should contact the corresponding author for more information.

## Declaration of interests

YQ and FZ report grants from GlaxoSmithKline Biologicals, Merck & Co, and Xiamen Innovax Biotech to their institution, to undertake clinical trials on the human papillomavirus (HPV) vaccine. MJ has previously received research grants (unrelated to this paper) from the National Institute for Health Research, Research Councils UK, Gavi, the Vaccine Alliance, the European Commission, and Wellcome Trust. MJ and YL have received research grants (related to this paper) from the Bill & Melinda Gates Foundation. Other co-authors declare no competing interests.
