# Supplementary material for: Cost-effectiveness analysis of switching from a bivalent to a nonavalent HPV vaccination programme in China: a modelling study
Source: Lancet Reg Health West Pac. 2025 Feb 20;56:101499. doi: 10.1016/j.lanwpc.2025.101499 (PMC11889945; doi:10.1016/j.lanwpc.2025.101499)
Supplement: Supplementary Figures and Tables [file mmc1.docx]

**Cost-effectiveness analysis of switching from a bivalent to a nonavalent HPV vaccination program in China: a modelling study**

**Supplementary appendix**

Contents

[**1. Methods** 2](#_Toc149332642)

[**1.1 Model structure** 2](#_Toc149332643)

[**1.2 Model assumptions and inputs** 3](#_Toc149332644)

[**1.2.1 Vaccine prices** 3](#_Toc149332645)

[**1.2.2 Treatment costs** 3](#_Toc149332646)

[**1.3 Demographic and epidemiological data** 6](#_Toc149332647)

[**1.4 Alternative vaccination strategies** 6](#_Toc149332648)

[**1.5 Screening scenarios** 6](#_Toc149332649)

[**2. Results** 9](#_Toc149332650)

[**2.1 Main results** 9](#_Toc149332651)

[**2.2 Sensitivity analysis** 16](#_Toc149332652)

# **1. Methods**

## **1.1 Model structure**

The transmission dynamic model in this study consists of a dynamic model and a natural history model, and the model structure is shown in Figure S1. The dynamic model was used to simulate the human papillomavirus (HPV) transmission between males and females, whereas the natural history model was employed to simulate the natural history of cervical cancer and to obtain the number of cervical cancer cases and deaths associated with HPV infections.

In the dynamic model, the sexual mixing matrices for each stratum were calculated by use of partner acquisition rates and assortativity of age, area, and sexual activity.^1^ Partner acquisition rates were adjusted to maintain the number of male-female sexual partnerships.^1^ We incorporated 13 HPV strains (types 16, 18, 31, 33, 35, 39, 45, 51, 52, 56, 58, 59, and 68), which were high-risk for the development of cervical cancer and its precursor lesions. The force of infection (FOI) and prevalence for every high-risk HPV type were simulated separately, because of the potential variations in transmission probability between every sexual pair and the substantial differences in vaccine efficacy for each high-risk HPV type. Natural immunity would be acquired for a period of time after an HPV type was cleared, however, this infection-acquired immunity would wane over time and only protected against reinfection with the same HPV type, without cross-protection against other HPV types.

In the natural history model, neonates are assumed to be in the uninfected state at birth, and all-cause mortality was present in all states. Each individual was entered into the model at their birth year if they were born after 2015 or at their age in 2015, and they were then transitioned from the old state to a new state within the model based on the transition probabilities. Individuals were transitioned between states representing no current or previous infection or vaccine (in which they were considered susceptible to infections); immunity (including infection-acquired immunity and vaccine-acquired immunity); infection; development of cervical intraepithelial neoplasia (CIN) grade 1, CIN2, or CIN3 disease (representing mild, moderate, and severe dysplasia); effective detection and treatment; and cervical cancer likely to cause death or to extend until the simulation end point.^1^ Local cervical cancer without symptoms may become symptomatic or progress to more advanced stages of cervical cancer without symptoms. In the absence of screening, cervical cancer is diagnosed only when symptoms develop in which the patient is immediately treated. Women with symptomatic cervical cancer are subjected to the age-specific death and cure probability of cervical cancer. Cancer patients who remain alive 5 years after cancer diagnosis are moved to the health state “cured” and are removed from the model in the rest of simulations.^2^

Vaccination and screening interventions were embedded into the model at different simulation stages. If HPV vaccination is available, girls aged 12 years were considered to be vaccinated at predefined coverage in the dynamic model, and vaccinated women acquired lifelong immunity based on vaccine efficacy. If cervical cancer screening is available, women at the target age for screening and without a diagnosis of cervical cancer were considered to be screened at predefined coverage in the natural history model. HPV infection, CIN, and cervical cancer will be detected based on the sensitivity of the screening scenario.


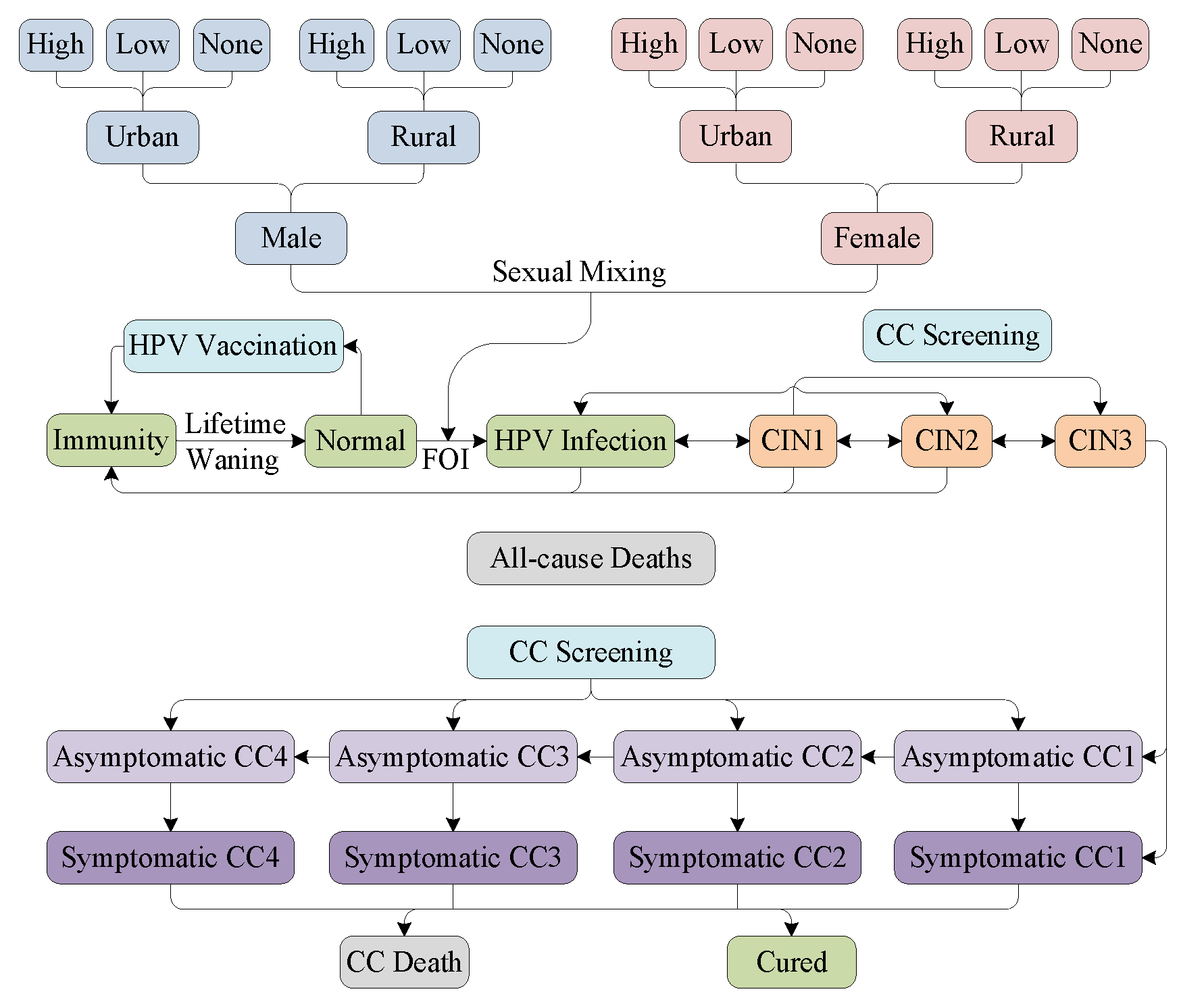


**Figure S1. Model structure**

Individuals with multiple sexual partners are regarded as having high sexual activity level, while individuals with only one sexual partner are regarded as having low sexual activity level.

Abbreviations: HPV, human papillomavirus; FOI, force of infection; CIN, cervical intraepithelial neoplasia; CC, cervical cancer.

## **1.2 Model assumptions and inputs**

## **1.2.1 Vaccine prices**

The current private market prices of HPV vaccines in China are $49·10, $119·10, and $193·73 per dose for domestic 2vHPV, 4vHPV, and 9vHPV vaccines, respectively. In the national vaccination program, China could likely negotiate lower public sector prices as part of a government tender. We assumed the base case price for 4vHPV vaccine based on the price paid by the Pan American Health Organization (PAHO) Revolving Fund in 2022,^3^ given that China is at a similar stage of economic development to many large PAHO countries^4^ and has bargaining power from a large potential market size. Due to the limited price information of the other three vaccine types, we calculate them using the 4vHPV vaccine base case price ($10·48 per dose) as reference and applying the ratio of prices for each vaccine in the current private market in China. Therefore, domestic 2vHPV and 9vHPV vaccines were estimated to cost $4·32 and $17·05 per dose, respectively.

## **1.2.2 Treatment costs**

The treatment cost for CIN and cervical cancer cases were collected in our nationwide multicenter cross-sectional, hospital-based survey. The study was conducted from August 2020 to June 2021, including 26 hospitals in seven administrative regions of China^5^. All components of direct medical costs, direct non-medical costs, and indirect costs (productivity loss) for women diagnosed with CIN and invasive cervical cancer were included.

Model parameters were obtained from published literature and government-released online datasets, which were summarised in Table S1. The range of values tested in the sensitivity analysis for each parameter was either based on the reported 95% confidence intervals in the referenced studies or determined by assuming a 25% change from the base-case value.

**Table S1. Model inputs**

| **Parameter** | **Base case** | **Range** | **Distribution** | **Source** |  |
| --- | --- | --- | --- | --- | --- |
| **Annual probability of HPV clearance** | |  |  |  | |
| 12-24 years | 0.7188 | 0.6463-0.7830 | β | ^6-9^ |  |
| 25-29 years | 0.6984 | 0.5898-0.7952 | β | ^6-9^ |  |
| 30-39 years | 0.3503 | 0.2860-0.4188 | β | ^6-9^ |  |
| 40-49 years | 0.2048 | 0.1118-0.3022 | β | ^6-10^ |  |
| >50 years | 0.1004 | 0.0546-0.1567 | β | ^6-9^ |  |
| **Annual probability of progression and regression** | |  |  |  | |
| Infection to CIN1 | 0.075 | 0.056-0.1154 | β | ^6,11^ |  |
| CIN1 to CIN2 | 0.2240 | 0.1608-0.2972 | β | ^6-9^ |  |
| CIN2 to CIN3 | 0.3498 | 0.0904-0.8654 | β | ^6-9^ |  |
| CIN3 to CC Ⅰ | 0.1019 | 0.0189-0.2906 | β | ^6-9^ |  |
| CIN2 to CIN1 | 0.2494 | 0.1994-0.2992 | β | ^6-9^ |  |
| CIN3 to CIN2 | 0.0135 | ±25% | β | ^6^ |  |
| CIN2 to infection/immunity | 0.1901 | ±25% | β | ^6,12^ |  |
| CIN1 to CIN3 | 0.0464 | 0.0098-0.1297 | β | ^6-9^ |  |
| CC Ⅰ to CC Ⅱ | 0.4376587 | ±25% | β | ^11,13^ |  |
| CC Ⅱ to CC Ⅲ | 0.5358411 | ±25% | β | ^11,13^ |  |
| CC Ⅲ to CC Ⅳ | 0.6837722 | ±25% | β | ^11,13^ |  |
| Waning of natural immunity | 0.021 | 0.015-0.027 | β | ^14^ |  |
| **Proportion of cancer symptoms** |  |  |  |  |  |
| CC Ⅰ | 0.15 | ±25% | β | ^11,13^ |  |
| CC Ⅱ | 0.225 | ±25% | β | ^11,13^ |  |
| CC Ⅲ | 0.6 | ±25% | β | ^11,13^ |  |
| CC Ⅳ | 0.9 | ±25% | β | ^11,13^ |  |
| **Vaccine efficacy for target types** |  |  |  |  |  |
| Full-course | 1.00 | 0.90-1.00 | Binomial | Assumed^15-17^ |  |
| Single dose | 0.85 | 0.80-0.90 | Binomial | Assumed |  |
| **Screening sensitivity** |  |  |  |  |  |
| **Liquid-based cytology** |  |  |  |  |  |
| CIN1 | 0.47 | ±25% | β | ^18^ |  |
| CIN2/3 | 0.53 | 0.486-0.574 | β | ^18^ |  |
| CC Ⅰ | 0.90 | ±25% | β | Assumed |  |
| CC Ⅱ | 0.92 | ±25% | β | Assumed |  |
| CC Ⅲ | 0.95 | ±25% | β | Assumed |  |
| CC Ⅳ | 0.99 | ±25% | β | Assumed |  |
| **HPV test** |  |  |  |  |  |
| CIN1 | 0.80 | ±25% | β | Assumed |  |
| CIN2 | 0.90 | ±25% | β | Assumed^17^ |  |
| CIN3 | 0.94 | ±25% | β | Assumed^17^ |  |
| CC Ⅰ-Ⅳ | 1.00 | .. | .. | Assumed |  |
| **Proportion of precancerous lesions management** | |  |  |  | |
| **Urban** |  |  |  |  |  |
| Follow-up of CIN1 | 0.4631 | 0.4168-0.5094 | β | ^1^ |  |
| Treatment of CIN1 | 0.4421 | 0.3979-0.4863 | β | ^1^ |  |
| Treatment of CIN2/3 | 0.9533 | 0.8580-1.0000 | β | ^1^ |  |
| **Rural** |  |  |  |  |  |
| Follow-up of CIN1 | 0.3871 | 0.3484-0.4258 | β | ^1^ |  |
| Treatment of CIN1 | 0.4535 | 0.4082-0.4989 | β | ^1^ |  |
| Treatment of CIN2/3 | 0.8946 | 0.8051-0.9841 | β | ^1^ |  |
| **Treatment efficacy** |  |  |  |  |  |
| **Urban** |  |  |  |  |  |
| CIN1 treatment efficacy | 1.0000 | 0.9956-1.0000 | .. | ^1^ |  |
| CIN2/3 treatment efficacy | 0.9367 | 0.9278-0.9455 | β | ^1^ |  |
| **Rural** |  |  |  |  |  |
| CIN1 treatment efficacy | 0.9978 | 0.9943-1.0000 | β | ^1^ |  |
| CIN2/3 treatment efficacy | 0.9000 | 0.8846-0.9154 | β | ^1^ |  |
| **Costs (2022 US$)** |  |  |  |  |  |
| **Vaccine price per dose** |  |  |  |  |  |
| Domestic 2vHPV vaccine | 4.32 | ±25% | γ | Assumed |  |
| 9vHPV vaccine | 17.05 | ±25% | γ | Assumed |  |
| **Vaccination service cost** | 4.02 | ±25% | γ | ^19^ |  |
| **Screening and treatment costs** |  |  |  |  |  |
| **Urban** |  |  |  |  |  |
| LBC-based screening | 10.07 | ±25% | γ | ^1^ |  |
| HPV-based screening | 15.81 | ±25% | γ | ^2^ |  |
| CIN1 treatment | 1728.18 | 1009.24-2816.67 | γ | ^5^ |  |
| CIN2/3 treatment | 2603.40 | 1666.34-3970.16 | γ | ^5^ |  |
| CC Ⅰ treatment | 18189.06 | 13266.27-25398.95 | γ | ^5^ |  |
| CC Ⅱ treatment | 26330.99 | 18068.71-40100.46 | γ | ^5^ |  |
| CC Ⅲ treatment | 27693.53 | 18159.25-41234.49 | γ | ^5^ |  |
| CC Ⅳ treatment | 29991.76 | 18856.24-53520.54 | γ | ^5^ |  |
| **Rural** |  |  |  |  |  |
| LBC-based screening | 7.31 | ±25% | γ | ^1^ |  |
| HPV-based screening | 13.56 | ±25% | γ | ^2^ |  |
| CIN1 treatment | 484.36 | 176.97-1403.8 | γ | ^5^ |  |
| CIN2/3 treatment | 1298.48 | 591.57-2220.77 | γ | ^5^ |  |
| CC Ⅰ treatment | 18189.06 | 13266.27-25398.95 | γ | ^5^ |  |
| CC Ⅱ treatment | 26330.99 | 18068.71-40100.46 | γ | ^5^ |  |
| CC Ⅲ treatment | 27693.53 | 18159.25-41234.49 | γ | ^5^ |  |
| CC Ⅳ treatment | 29991.76 | 18856.24-53520.54 | γ | ^5^ |  |
| **Utilities (quality of life)** | |  |  |  | |
| 6 month posttreatment of CIN1 | 0.97 | ±25% | β | ^20^ |  |
| pretreatment of CIN2/3 | 0.90 | 0.8734-0.9266 | β | ^21^ |  |
| 1 month posttreatment of CIN2/3 | 0.96 | 0.9445-0.9755 | β | ^21^ |  |
| 3 month posttreatment of CIN2/3 | 0.98 | 0.9445-0.9944 | β | ^21^ |  |
| 6 month posttreatment of CIN2/3 | 0.99 | 0.9856-0.9944 | β | ^21^ |  |
| pretreatment of CC Ⅰ-Ⅱa | 0.83 | 0.7875-0.8725 | β | ^21^ |  |
| 1 month posttreatment of CC Ⅰ-Ⅱa | 0.77 | 0.7232-0.8168 | β | ^21^ |  |
| 3 month posttreatment of CC Ⅰ-Ⅱa | 0.84 | 0.7975-0.8825 | β | ^21^ |  |
| 6 month posttreatment of CC Ⅰ-Ⅱa | 0.94 | 0.9145-0.9655 | β | ^21^ |  |
| pretreatment of CCⅡb-Ⅳ | 0.84 | 0.7731-0.9069 | β | ^21^ |  |
| 1 month posttreatment of CC Ⅱb-Ⅳ | 0.68 | 0.5674-0.7926 | β | ^21^ |  |
| 3 month posttreatment of CC Ⅱb-Ⅳ | 0.75 | 0.6409-0.8591 | β | ^21^ |  |
| 6 month posttreatment of CC Ⅱb-Ⅳ | 0.86 | 0.8213-0.8987 | β | ^21^ |  |
| terminal care | 0.288 | ±25% | β | ^20^ |  |
| **Achievable coverage of vaccination** | 0.90 | 0.70-0.95 | Binomial | Assumed^22^ |  |
| **Achievable coverage of screening** | 0.90 | 0.70-0.90 | Binomial | ^22,23^ |  |
| **Discount rate** | 0.03 | 0-0.05 | Binomial | ^24^ |  |

Abbreviations: CIN, cervical intraepithelial neoplasia; CC, cervical cancer; LBC, liquid-based cytology; HPV, Human Papillomavirus.

Urban population refers to all people residing in urban areas, while rural population refers to people residing in rural areas.

## **1.3 Demographic and epidemiological data**

Demographic and epidemiological data were obtained from open-source publications or government-released online datasets^25-32^ and were reported in the previous study.^2^ Population size in 2015 was obtained from National Bureau of Statistics of China.^25,26^ The all-cause mortality rate was obtained from National Health Commission of China.^27^ Age-specific incidence and mortality of cervical cancer in 2015 were obtained from Chinese Cancer Registry Report released by National Cancer Center of China.^28^ In addition, the data sources and analytical methods of HPV prevalence, fertility rate, sexual activity, and screening coverage were reported in the previous study.^1^ The projected fertility rate of women aged 15-49 years^29^ and urbanisation data^30,31^ were from the UN Population Division. Proportion of high sexual activity population (residents who have multiple sexual partners lifetime) from 2000 to 2015 was obtained from a national representative longitudinal survey in China. Based on the trends from 2000 to 2015, sexual activity in China is expected to substantially increase in the coming decades. We assume that the upward trends will continue until the year when the values reach the targets, then a constant trend will maintain to 2100. Due to the scarce data on the prediction of sexual activity in China, the patterns in sexual activity in China were projected with historical data from the USA as a reference and were adjusted for the Human Development Index.^32^

## **1.4 Alternative vaccination strategies**

This study included five same-vaccine strategies and two mixed-vaccine strategies. The details of vaccination strategies were described in Figure S2 and S3.

## **1.5 Screening scenarios**

The current age-specific screening coverage was derived from a nationwide survey (Figure S4).^33,34^ The screening scenarios were assumed to involve liquid-based cytology (LBC)-based and HPV-based screening. For LBC-based screening scenarios, women with atypical squamous cell of undetermined significance (ASC-US+) will be referred to colposcopy. Women with negative cytology will recall for routine screening at 3-year intervals. The sensitivity of LBC-based screening scenario was 0·47 to detect CIN1, and 0·53 to detect CIN2/3 (Table S1). HPV-based screening scenario used HPV DNA testing as primary screening (with genotyping) and cytology triage, namely women positive for HPV16/18 will be referred to colposcopy and women positive for other 11 oncogenic types will be triaged with LBC. Women negative for HPV test will recall for routine screening at 5-year intervals. The sensitivity of HPV-based screening scenario was 0·80, 0·90, and 0·94 to detect CIN1, CIN2, and CIN3, respectively (Table S1).

The proportion of screening women in compliance with routine cervical screening is estimated from the national wide expert panel review. The results suggested that about 95·33% of urban women and 89·46% of rural women will receive treatment after the diagnosis of CIN2/3. As to CIN1, about 44·21% of urban women, and 45·35% of rural women will receive treatment (Table S1). Among the women who receive treatment, about 100% and 93·70% of urban women, and 99·80% and 90·00% of rural women were successfully treated after the treatment of CIN1 and CIN2/3, respectively (Table S1).

**Figure S2. Description of four same-vaccine strategies with the same vaccine for all doses**

**Figure S3. Description of two mixed-vaccine strategies with one dose of 2vHPV and one dose of 9vHPV**


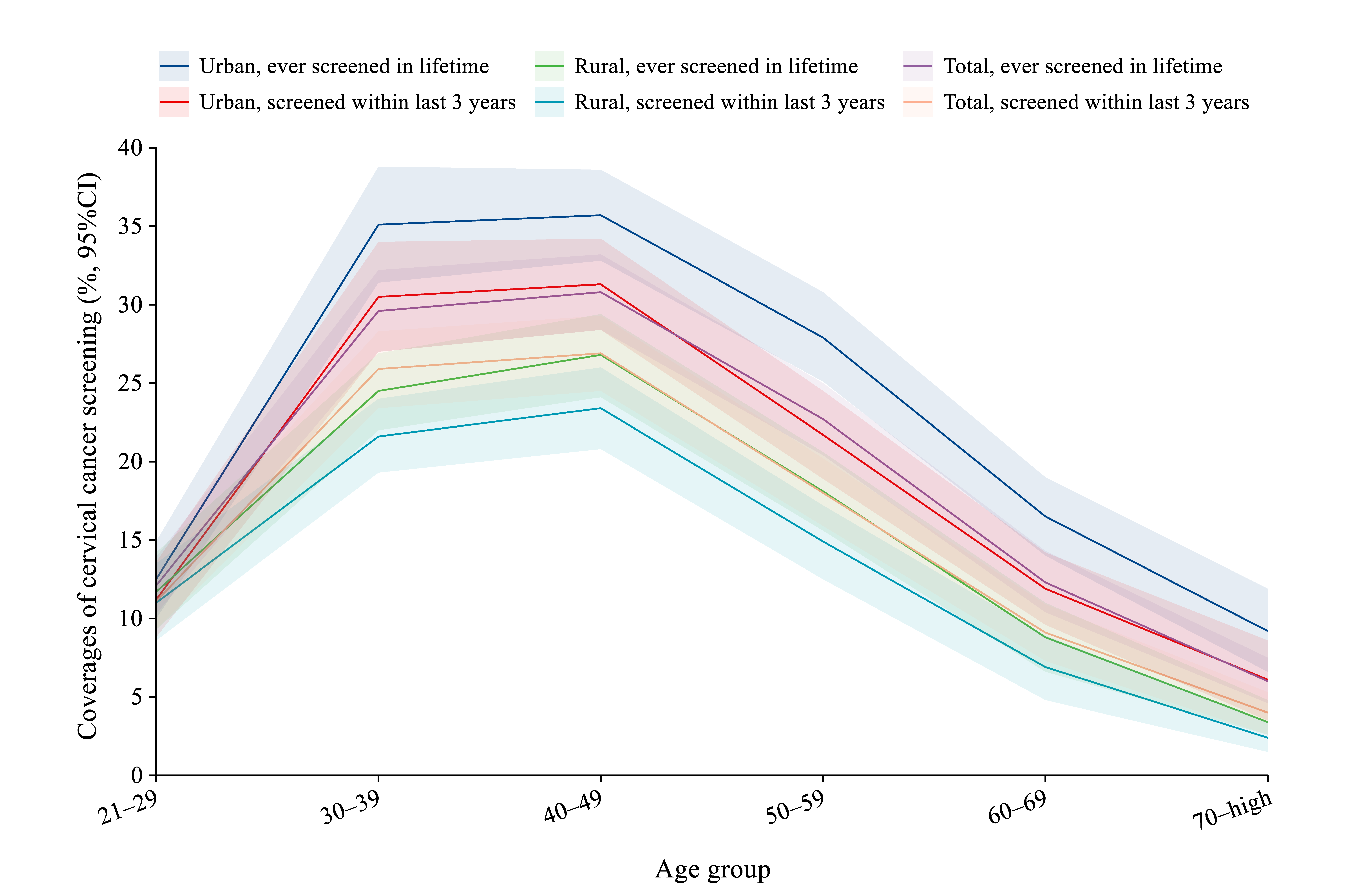


**Figure S4. Current cervical cancer screening coverage**

# **2. Results**

## **2.1 Main results**

The cost-effectiveness frontier of alternative vaccination strategies under the improved HPV-based screening scenario is shown in Figure S5. The details of cases averted, deaths averted, costs saved, and QALYs gained in different vaccination strategies compared with no vaccination are shown in Tables S2-S3 and Figure S6. In the same-vaccine strategies, regardless of the screening scenarios, 9vHPV vaccination with catch-up was the only dominant strategy if domestic 9vHPV vaccine was available before 2030 and switching from 2vHPV to 9vHPV vaccination was the only dominant strategy if domestic 9vHPV vaccine was available after 2035. In all same-vaccine and mixed-vaccine strategies, regardless of the screening scenarios and the year when domestic 9vHPV vaccine was available, the mixed-vaccine strategy without restricted intervals was the only dominant strategy. The effect of vaccination age at the second dose on health and economic benefits of the mixed-vaccine strategy is shown in Figure S7. The number and proportion of vaccinated women who received one dose of 2vHPV and one dose of 9vHPV vaccine in alternative strategies are shown in Figure S8.

**Table S2. Cost-effectiveness analysis of different HPV vaccination strategies under status quo screening**

| 9vHPV initiation year | Vaccination strategies | Cases averted | Deaths averted | Total discounted costs ($ million) | Cost saving ($ million) | QALYs gained | Cost-effectiveness of same-vaccine strategies | Cost-effectiveness of all strategies |
| --- | --- | --- | --- | --- | --- | --- | --- | --- |
| 2030 | No vaccination | 0 | 0 | 146,837 | 0 | 0 | Dominated | Dominated |
|  | Maintaining 2vHPV | 6,816,890 | 2,381,567 | 129,869 | 16,969 | 4,806,054 | Dominated | Dominated |
|  | Waiting for 9vHPV | 9,565,430 | 3,499,561 | 129,046 | 17,791 | 5,146,541 | Dominated | Dominated |
|  | 2vHPV to 9vHPV | 10,349,199 | 3,775,045 | 124,657 | 22,180 | 6,374,297 | Dominated | Dominated |
|  | 9vHPV + catch-up | 10,560,317 | 3,864,861 | 124,408 | 22,429 | 6,569,433 | Cost-saving* | Dominated |
|  | Mixed-vaccine strategy (no interval restriction) | 10,692,598 | 3,912,454 | 122,790 | 24,047 | 6,788,168 | - | Cost-saving* |
|  | Mixed-vaccine strategy (5y-interval) | 10,588,653 | 3,870,839 | 123,390 | 23,447 | 6,655,484 | - | Dominated |
| 2035 | No vaccination | 0 | 0 | 146,837 | 0 | 0 | Dominated | Dominated |
|  | Maintaining 2vHPV | 6,816,890 | 2,381,567 | 129,869 | 16,969 | 4,806,054 | Dominated | Dominated |
|  | Waiting for 9vHPV | 8,804,472 | 3,220,099 | 132,306 | 14,531 | 4,208,440 | Dominated | Dominated |
|  | 2vHPV to 9vHPV | 10,089,222 | 3,671,040 | 125,534 | 21,303 | 6,107,190 | Cost-saving* | Dominated |
|  | 9vHPV + catch-up | 9,729,799 | 3,559,809 | 128,541 | 18,297 | 5,365,706 | Dominated | Dominated |
|  | Mixed-vaccine strategy (no interval restriction) | 10,685,548 | 3,910,061 | 122,395 | 24,442 | 6,770,901 | - | Cost-saving* |
|  | Mixed-vaccine strategy (5y-interval) | 10,320,344 | 3,763,490 | 124,425 | 22,412 | 6,344,184 | - | Dominated |
| 2040 | No vaccination | 0 | 0 | 146,837 | 0 | 0 | Dominated | Dominated |
|  | Maintaining 2vHPV | 6,816,890 | 2,381,567 | 129,869 | 16,969 | 4,806,054 | Dominated | Dominated |
|  | Waiting for 9vHPV | 8,129,039 | 2,971,694 | 134,798 | 12,039 | 3,487,097 | Dominated | Dominated |
|  | 2vHPV to 9vHPV | 9,851,518 | 3,575,917 | 126,230 | 20,607 | 5,895,360 | Cost-saving* | Dominated |
|  | 9vHPV + catch-up | 8,949,077 | 3,273,235 | 131,920 | 14,917 | 4,375,910 | Dominated | Dominated |
|  | Mixed-vaccine strategy (no interval restriction) | 10,662,401 | 3,902,921 | 122,201 | 24,636 | 6,718,464 | - | Cost-saving* |
|  | Mixed-vaccine strategy (5y-interval) | 10,062,771 | 3,660,444 | 125,320 | 21,517 | 6,083,147 | - | Dominated |
| 2045 | No vaccination | 0 | 0 | 146,837 | 0 | 0 | Dominated | Dominated |
|  | Maintaining 2vHPV | 6,816,890 | 2,381,567 | 129,869 | 16,969 | 4,806,054 | Dominated | Dominated |
|  | Waiting for 9vHPV | 7,502,785 | 2,741,498 | 136,777 | 10,061 | 2,909,924 | Dominated | Dominated |
|  | 2vHPV to 9vHPV | 9,626,783 | 3,486,251 | 126,795 | 20,042 | 5,722,098 | Cost-saving* | Dominated |
|  | 9vHPV + catch-up | 8,258,641 | 3,019,353 | 134,504 | 12,333 | 3,616,812 | Dominated | Dominated |
|  | Mixed-vaccine strategy (no interval restriction) | 10,591,784 | 3,879,722 | 122,413 | 24,425 | 6,594,392 | - | Cost-saving* |
|  | Mixed-vaccine strategy (5y-interval) | 9,826,516 | 3,565,927 | 126,024 | 20,813 | 5,875,632 | - | Dominated |
| 2050 | No vaccination | 0 | 0 | 146,837 | 0 | 0 | Dominated | Dominated |
|  | Maintaining 2vHPV | 6,816,890 | 2,381,567 | 129,869 | 16,969 | 4,806,054 | Dominated | Dominated |
|  | Waiting for 9vHPV | 6,889,681 | 2,516,653 | 138,442 | 8,395 | 2,422,716 | Dominated | Dominated |
|  | 2vHPV to 9vHPV | 9,404,004 | 3,397,810 | 127,279 | 19,558 | 5,573,496 | Cost-saving* | Dominated |
|  | 9vHPV + catch-up | 7,625,722 | 2,786,644 | 136,540 | 10,298 | 3,016,013 | Dominated | Dominated |
|  | Mixed-vaccine strategy (no interval restriction) | 10,464,842 | 3,836,384 | 122,999 | 23,838 | 6,406,273 | - | Cost-saving* |
|  | Mixed-vaccine strategy (5y-interval) | 9,602,006 | 3,476,395 | 126,590 | 20,247 | 5,705,140 | - | Dominated |

Abbreviations: HPV, Human Papillomavirus; 2vHPV, bivalent HPV vaccine; 9vHPV, nonavalent HPV vaccine

*Non-dominated strategies are cost-saving.

When reporting cost-effectiveness, we indicate strategies that are more costly and less effective than an alternative strategy as “dominated”, and indicate non-dominated strategies that are cost-saving as “Cost-saving”.

**Table S3. Cost-effectiveness analysis of different HPV vaccination strategies under improved HPV-based screening**

| 9vHPV initiation year | Vaccination strategies | Cases averted | Deaths averted | Total discounted costs ($ million) | Cost saving ($ millions) | QALYs gained | Cost-effectiveness of same-vaccine strategies | Cost-effectiveness of all strategies |
| --- | --- | --- | --- | --- | --- | --- | --- | --- |
| 2030 | No vaccination | 0 | 0 | 135,883 | 0 | 0 | Dominated | Dominated |
|  | Maintaining 2vHPV | 3,168,782 | 978,856 | 125,205 | 10,678 | 1,994,722 | Dominated | Dominated |
|  | Waiting for 9vHPV | 3,882,666 | 1,250,181 | 124,184 | 11,699 | 1,976,689 | Dominated | Dominated |
|  | 2vHPV to 9vHPV | 4,246,120 | 1,363,961 | 121,416 | 14,467 | 2,476,689 | Dominated | Dominated |
|  | 9vHPV + catch-up | 4,290,279 | 1,383,256 | 121,207 | 14,676 | 2,515,004 | Cost-saving* | Dominated |
|  | Mixed-vaccine strategy (no interval restriction) | 4,349,526 | 1,402,258 | 119,872 | 16,012 | 2,602,307 | - | Cost-saving* |
|  | Mixed-vaccine strategy (5y-interval) | 4,317,952 | 1,390,585 | 120,364 | 15,519 | 2,561,815 | - | Dominated |
| 2035 | No vaccination | 0 | 0 | 135,883 | 0 | 0 | Dominated | Dominated |
|  | Maintaining 2vHPV | 3,168,782 | 978,856 | 125,205 | 10,678 | 1,994,722 | Dominated | Dominated |
|  | Waiting for 9vHPV | 3,573,887 | 1,149,546 | 126,366 | 9,517 | 1,621,347 | Dominated | Dominated |
|  | 2vHPV to 9vHPV | 4,168,464 | 1,335,242 | 122,086 | 13,797 | 2,395,873 | Cost-saving* | Dominated |
|  | 9vHPV + catch-up | 3,949,374 | 1,271,883 | 123,932 | 11,952 | 2,058,852 | Dominated | Dominated |
|  | Mixed-vaccine strategy (no interval restriction) | 4,345,990 | 1,401,235 | 119,469 | 16,414 | 2,590,970 | - | Cost-saving* |
|  | Mixed-vaccine strategy (5y-interval) | 4,237,568 | 1,360,805 | 121,162 | 14,721 | 2,467,337 | - | Dominated |
| 2040 | No vaccination | 0 | 0 | 135,883 | 0 | 0 | Dominated | Dominated |
|  | Maintaining 2vHPV | 3,168,782 | 978,856 | 125,205 | 10,678 | 1,994,722 | Dominated | Dominated |
|  | Waiting for 9vHPV | 3,299,235 | 1,060,128 | 128,018 | 7,865 | 1,345,042 | Dominated | Dominated |
|  | 2vHPV to 9vHPV | 4,096,841 | 1,308,870 | 122,604 | 13,279 | 2,331,264 | Cost-saving* | Dominated |
|  | 9vHPV + catch-up | 3,632,491 | 1,168,627 | 126,174 | 9,709 | 1,684,600 | Dominated | Dominated |
|  | Mixed-vaccine strategy (no interval restriction) | 4,335,372 | 1,398,726 | 119,240 | 16,643 | 2,564,396 | - | Cost-saving* |
|  | Mixed-vaccine strategy (5y-interval) | 4,160,552 | 1,332,334 | 121,853 | 14,030 | 2,388,289 | - | Dominated |
| 2045 | No vaccination | 0 | 0 | 135,883 | 0 | 0 | Dominated | Dominated |
|  | Maintaining 2vHPV | 3,168,782 | 978,856 | 125,205 | 10,678 | 1,994,722 | Dominated | Dominated |
|  | Waiting for 9vHPV | 3,043,373 | 977,131 | 129,311 | 6,572 | 1,121,383 | Dominated | Dominated |
|  | 2vHPV to 9vHPV | 4,028,349 | 1,283,893 | 123,010 | 12,873 | 2,277,841 | Cost-saving* | Dominated |
|  | 9vHPV + catch-up | 3,351,971 | 1,077,260 | 127,878 | 8,006 | 1,394,596 | Dominated | Dominated |
|  | Mixed-vaccine strategy (no interval restriction) | 4,312,792 | 1,393,273 | 119,348 | 16,535 | 2,521,469 | - | Cost-saving* |
|  | Mixed-vaccine strategy (5y-interval) | 4,089,270 | 1,306,112 | 122,381 | 13,502 | 2,324,926 | - | Dominated |
| 2050 | No vaccination | 0 | 0 | 135,883 | 0 | 0 | Dominated | Dominated |
|  | Maintaining 2vHPV | 3,168,782 | 978,856 | 125,205 | 10,678 | 1,994,722 | Dominated | Dominated |
|  | Waiting for 9vHPV | 2,792,663 | 896,201 | 130,392 | 5,491 | 931,786 | Dominated | Dominated |
|  | 2vHPV to 9vHPV | 3,960,093 | 1,259,257 | 123,353 | 12,530 | 2,231,762 | Cost-saving* | Dominated |
|  | 9vHPV + catch-up | 3,093,599 | 993,373 | 129,202 | 6,681 | 1,162,256 | Dominated | Dominated |
|  | Mixed-vaccine strategy (no interval restriction) | 4,278,399 | 1,384,631 | 119,765 | 16,118 | 2,465,342 | - | Cost-saving* |
|  | Mixed-vaccine strategy (5y-interval) | 4,020,796 | 1,281,167 | 122,791 | 13,092 | 2,272,322 | - | Dominated |

Abbreviations: HPV, Human Papillomavirus; 2vHPV, bivalent HPV vaccine; 9vHPV, nonavalent HPV vaccine

*Non-dominated strategies are cost-saving.

When reporting cost-effectiveness, we indicate strategies that are more costly and less effective than an alternative strategy as “dominated”, and indicate non-dominated strategies that are cost-saving as “Cost-saving”.

**
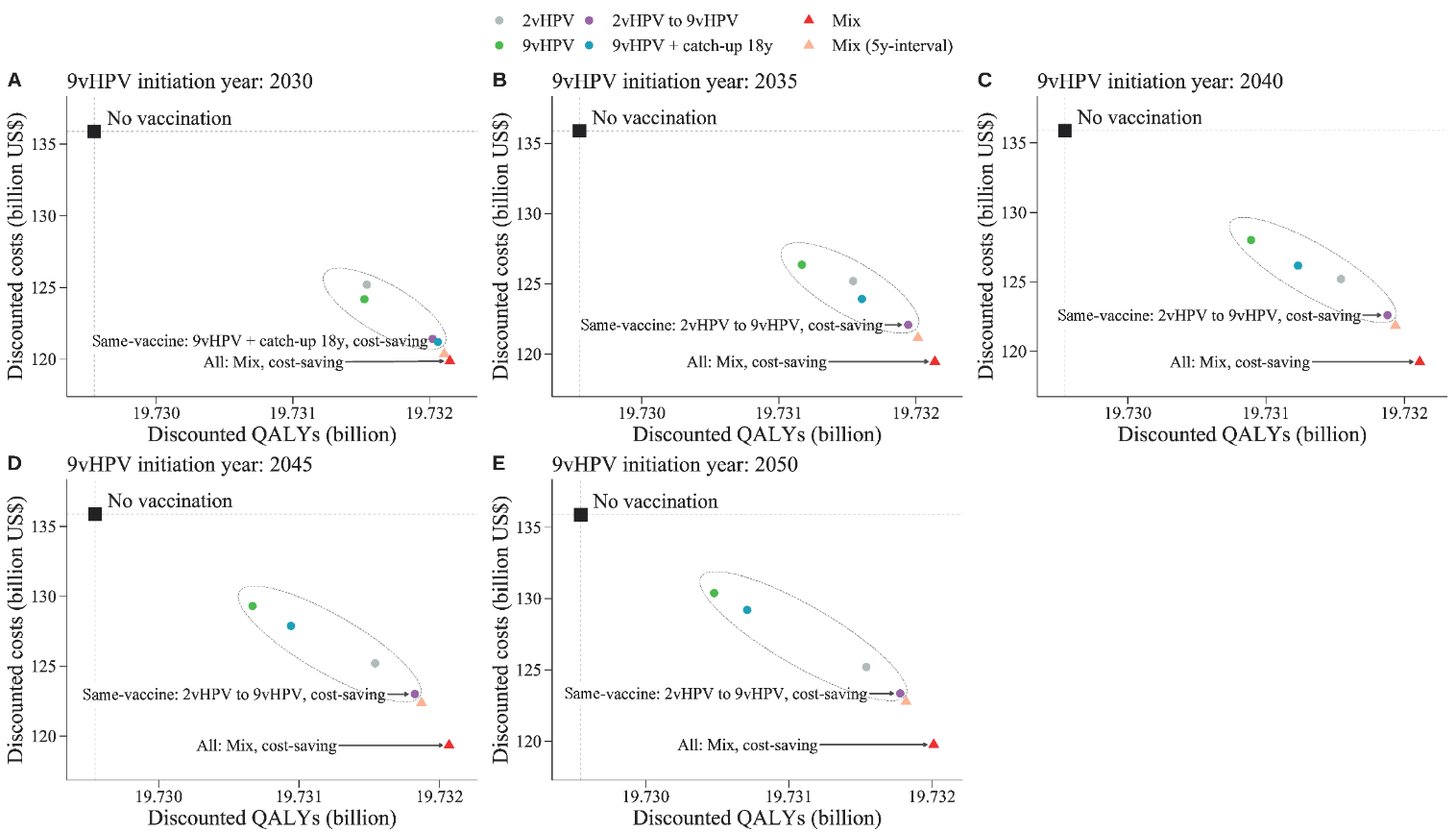
**

**Figure S5. Cost-effectiveness for different vaccination strategies compared with no vaccination under improved HPV-based screening, with 3% discounting.**

Abbreviations: HPV, Human Papillomavirus; 2vHPV, bivalent HPV vaccine; 9vHPV, nonavalent HPV vaccine; QALY, quality-adjusted life-year.

The black squares indicate the reference strategy of no vaccination. The circles inside dashed ovals indicate same-vaccine strategies, while the triangles indicate mixed-vaccine strategies. The “mix” strategy represents mixed two-dose schedule (first dose of 2vHPV vaccine targeting girls aged 12 and a second dose of 9vHPV vaccine in the 9vHPV initiation year) from 2023, and switching to two-dose routine 9vHPV vaccination when 9vHPV vaccine is available for national vaccination. The “mix (5y-interval)” represents the strategy that adds the restriction of a 5-year maximum interval between two doses based on the “mix” strategy. The strategies in the upper left quadrant are dominated by the strategies in the lower right quadrant. The labels represent the dominant vaccination strategy in the same-vaccine strategies and all strategies (including same-vaccine and mixed-vaccine strategies), respectively. Because the dominant strategies are cost-saving, the ICERs are negative and not shown.


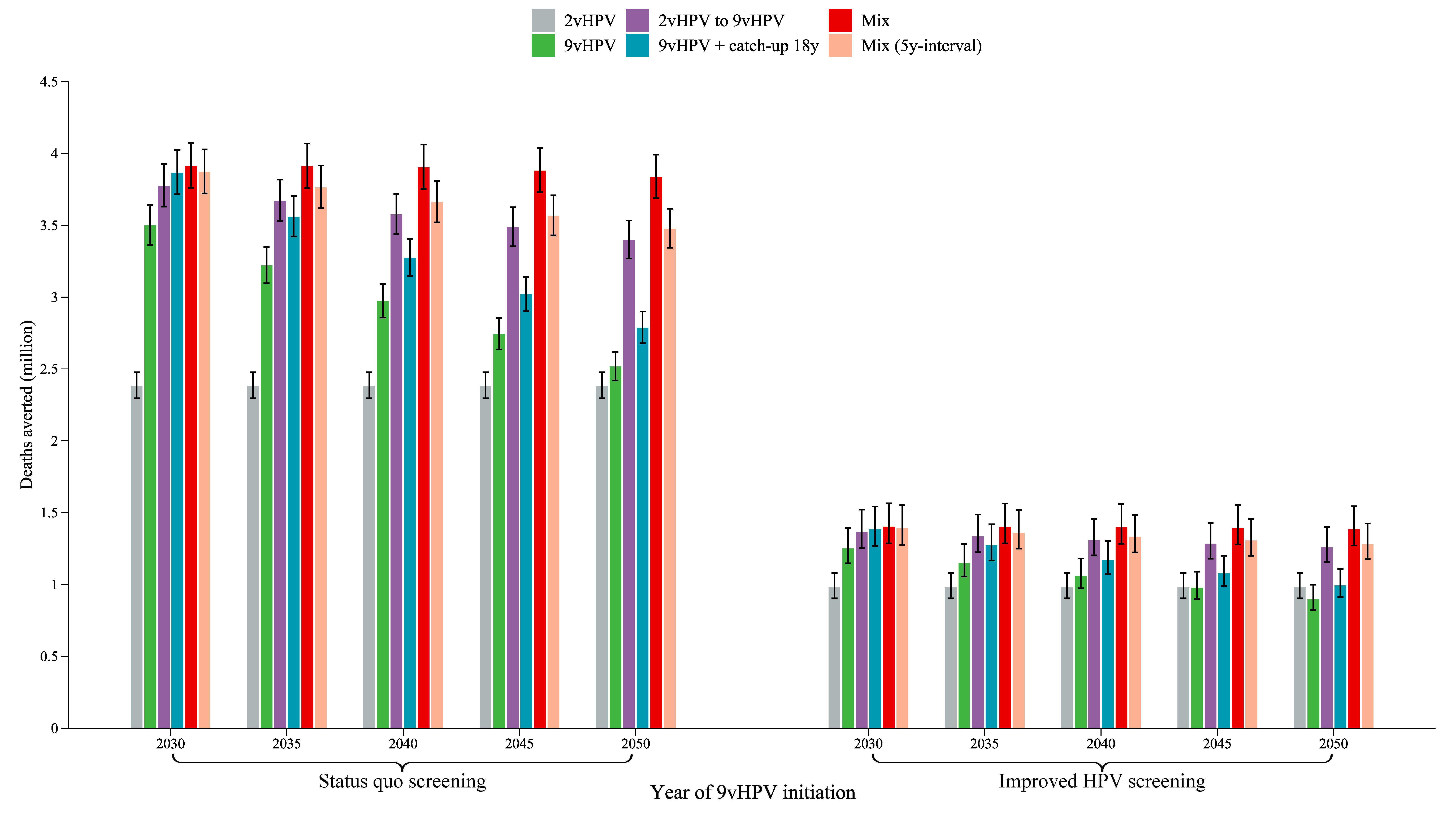


**Figure S6. Cervical cancer deaths averted of different vaccination strategies compared with no vaccination by 9vHPV initiation year and screening scenarios.**

Abbreviations: HPV, Human Papillomavirus; 2vHPV, bivalent HPV vaccine; 9vHPV, nonavalent HPV vaccine.

Error bars represent the 80% uncertainty intervals of cases averted compared with no vaccination. The “mix” strategy represents mixed two-dose schedule (first dose of 2vHPV vaccine targeting girls aged 12 and a second dose of 9vHPV vaccine in the 9vHPV initiation year) from 2023, and switching to two-dose routine 9vHPV vaccination when 9vHPV vaccine is available for national vaccination. The “mix (5y-interval)” represents the strategy that adds the restriction of a 5-year maximum interval between two doses based on the “mix” strategy. The improved HPV-based screening scenario represents switching to 5-yearly HPV-based screening in 2022, with linearly increasing age-specific uptake from status quo in 2021 to 70% in 2030, followed by a 1% increase every year till 90% is reached.


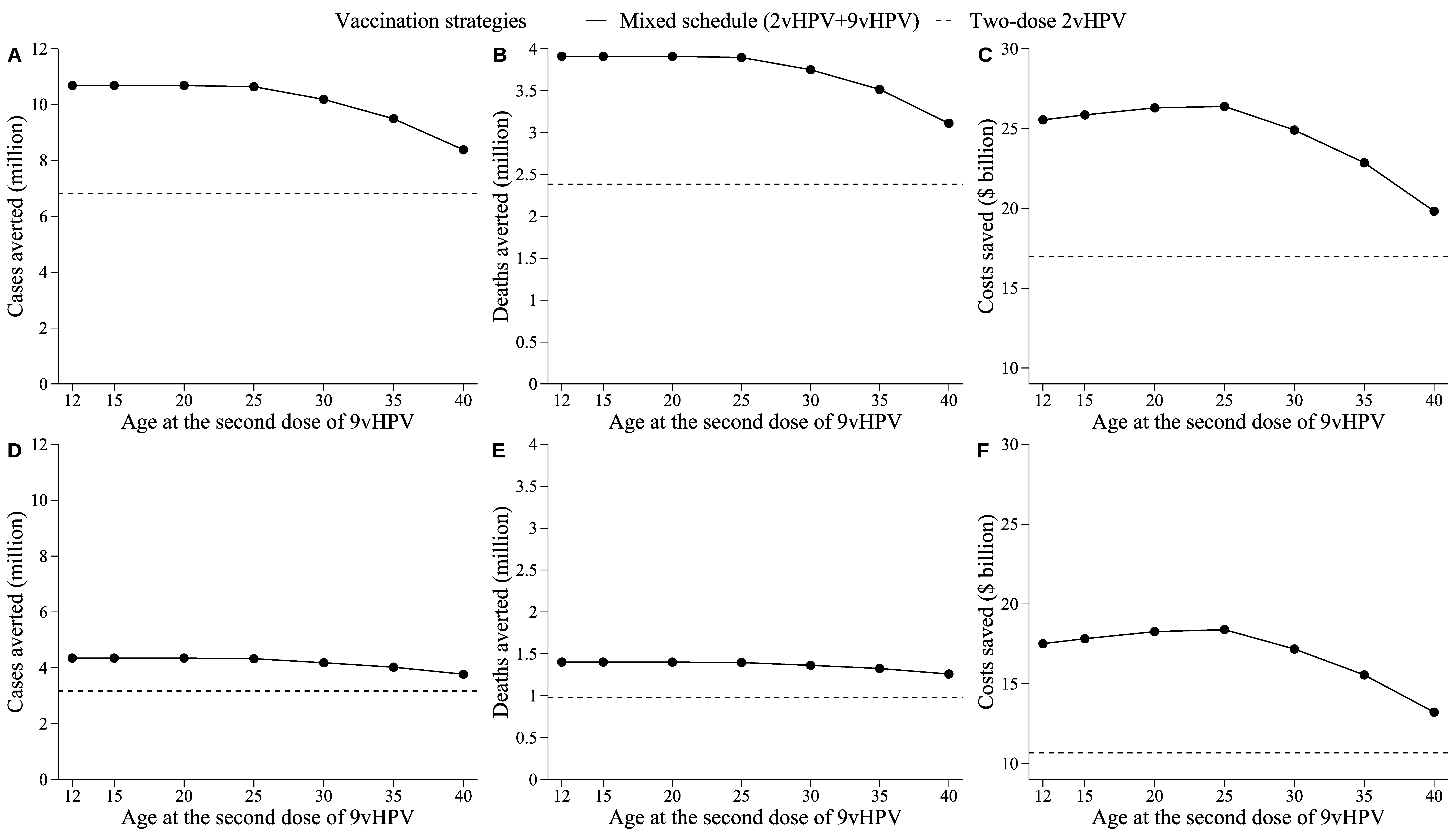


**Figure S7.** **Health and economic benefits of the mixed-vaccine strategy by vaccination age at the second dose under (A-C) status quo screening and (D-F) improved HPV-based screening.**

Abbreviations: HPV, Human Papillomavirus; 2vHPV, bivalent HPV vaccine; 9vHPV, nonavalent HPV vaccine.

**
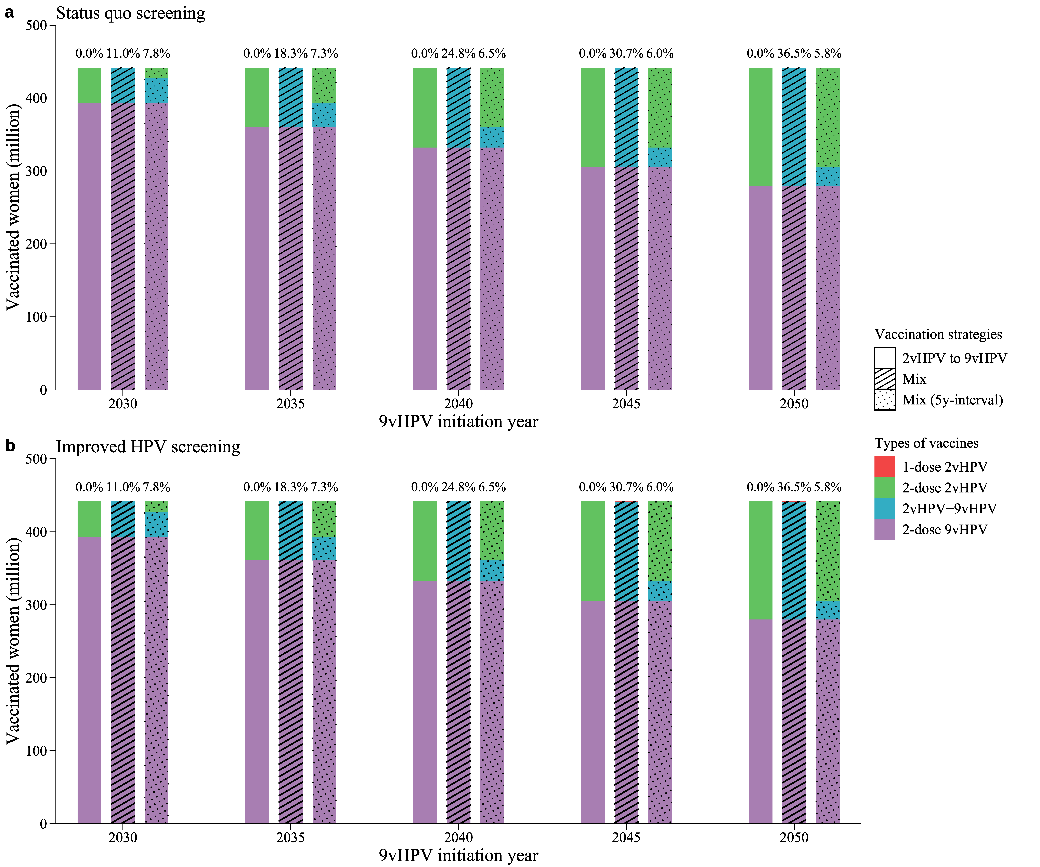
**

**Figure S8. Numbers of vaccinated women in different vaccination strategies.**

Abbreviations: HPV, Human Papillomavirus; 2vHPV, bivalent HPV vaccine; 9vHPV, nonavalent HPV vaccine.

The values above the bars represented the proportion of vaccinated women who received one dose of 2vHPV and one dose of 9vHPV vaccine (2vHPV+9vHPV) in each vaccination strategy over the analysis time horizon from 2023 to 2100, with all cohorts followed up until the end of their lives.

## **2.2 Sensitivity analysis**

The vaccination coverage of the second dose in mixed-vaccine strategies had little impact on the cost-effectiveness analysis (Figure S9-S10). When assuming current market vaccine prices, switching to 9vHPV vaccination and mixed-vaccine strategies were not cost-effective, thus the optimal strategy was maintaining 2vHPV vaccination (Figure S11-S12). The optimal strategies in same-vaccine strategies and all strategies did not change when using 3% and 0% discounting for costs and QALYs in the cost-effectiveness analysis (Figure S13-S14). Deterministic sensitivity analyses are displayed in a tornado diagram for model parameters (Figure S15-S18). In this diagram, each bar represents the impact of uncertainty in an individual variable. Probabilistic sensitivity analyses are displayed in scatter plots for discounted incremental costs and QALYs (Figure S19-S22).


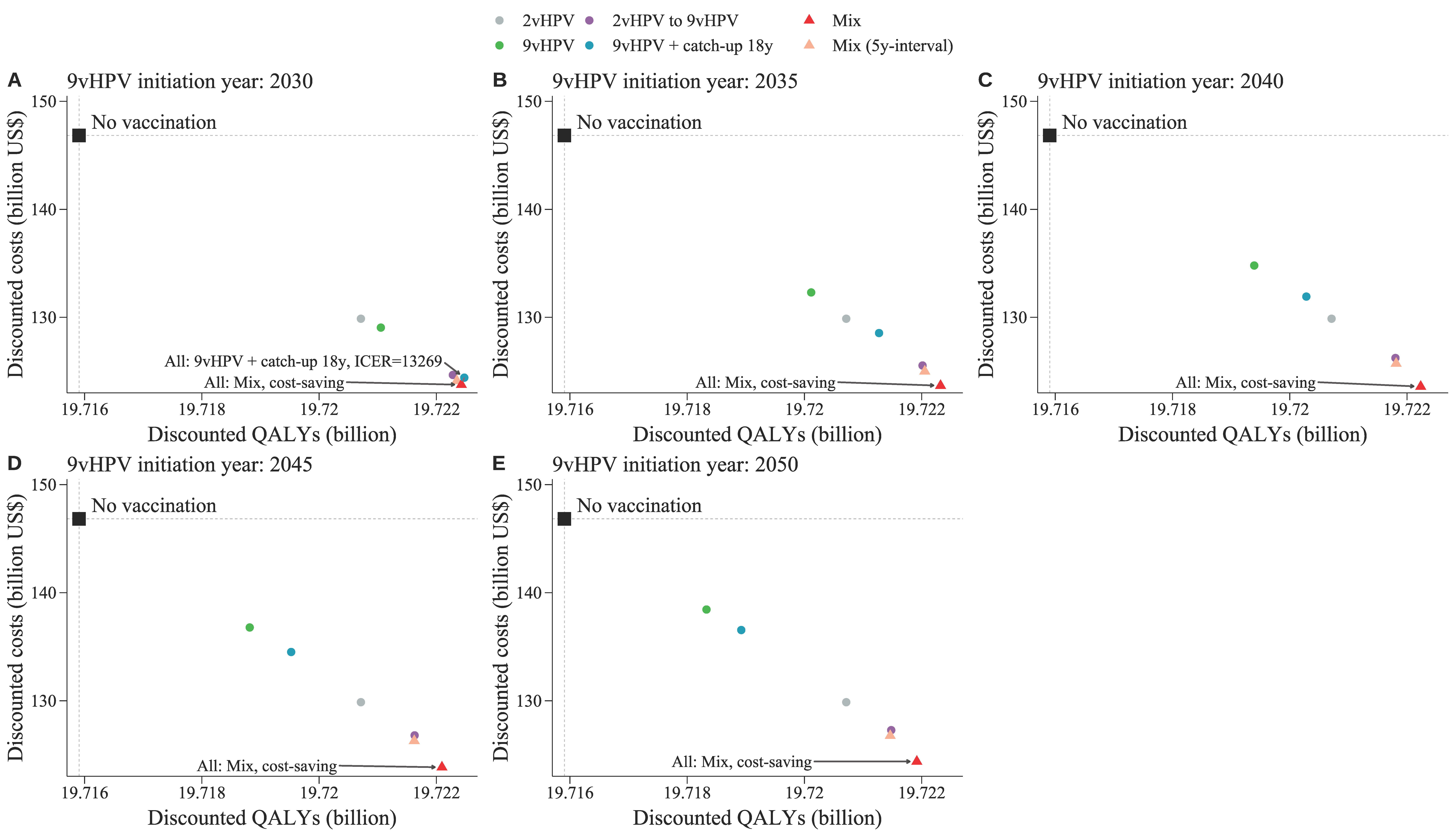


**Figure S9. Cost-effectiveness for different vaccination strategies under status quo screening assuming 40% vaccination coverage of the second dose for the mixed-vaccine strategy**

Abbreviations: HPV, Human Papillomavirus; 9vHPV, nonavalent HPV vaccine; QALY, quality-adjusted life-year.


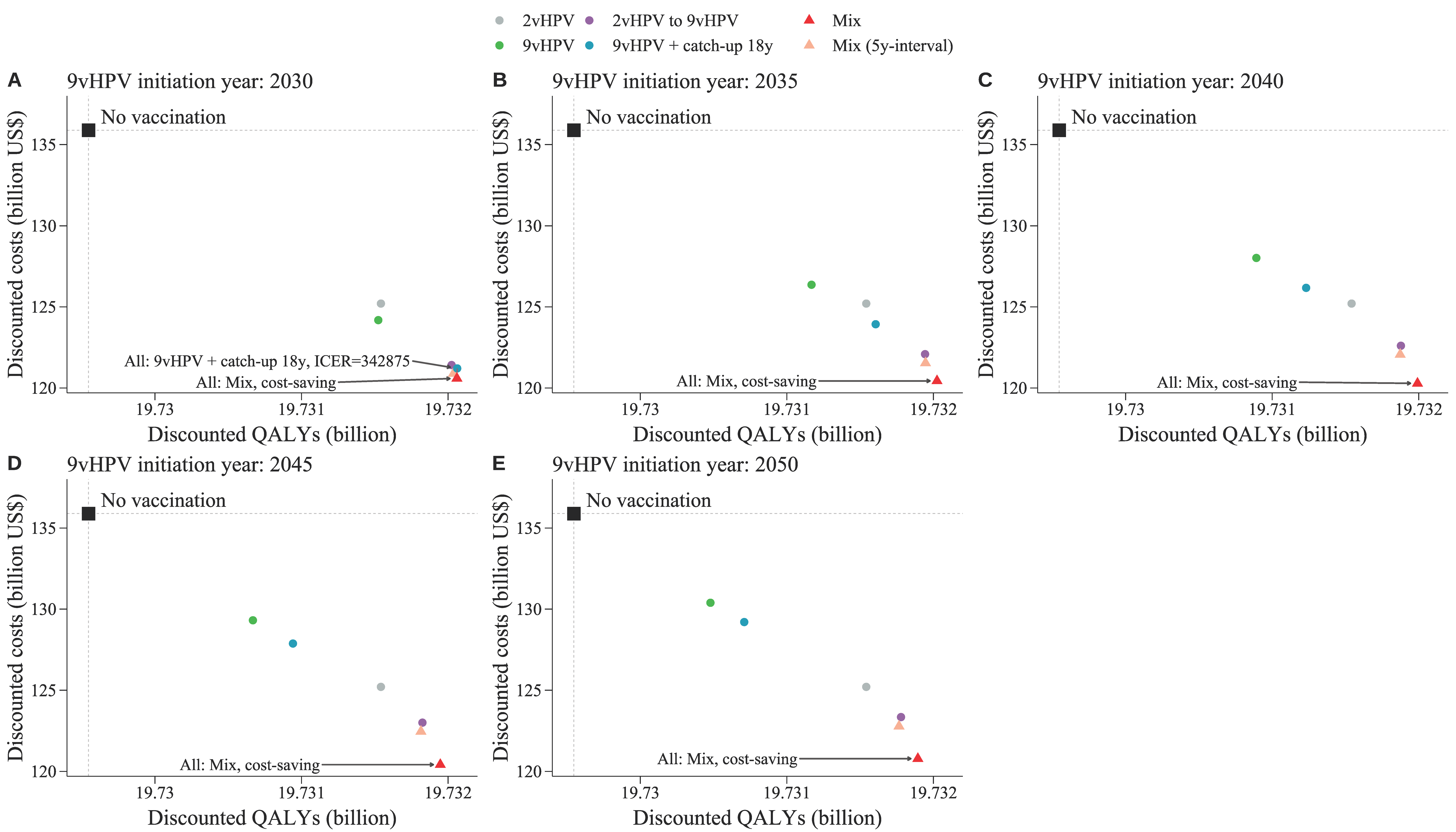


**Figure S10. Cost-effectiveness for different vaccination strategies under improved HPV-based screening assuming 40% vaccination coverage of the second dose for the mixed-vaccine strategy**

Abbreviations: HPV, Human Papillomavirus; 9vHPV, nonavalent HPV vaccine; QALY, quality-adjusted life-year.

**
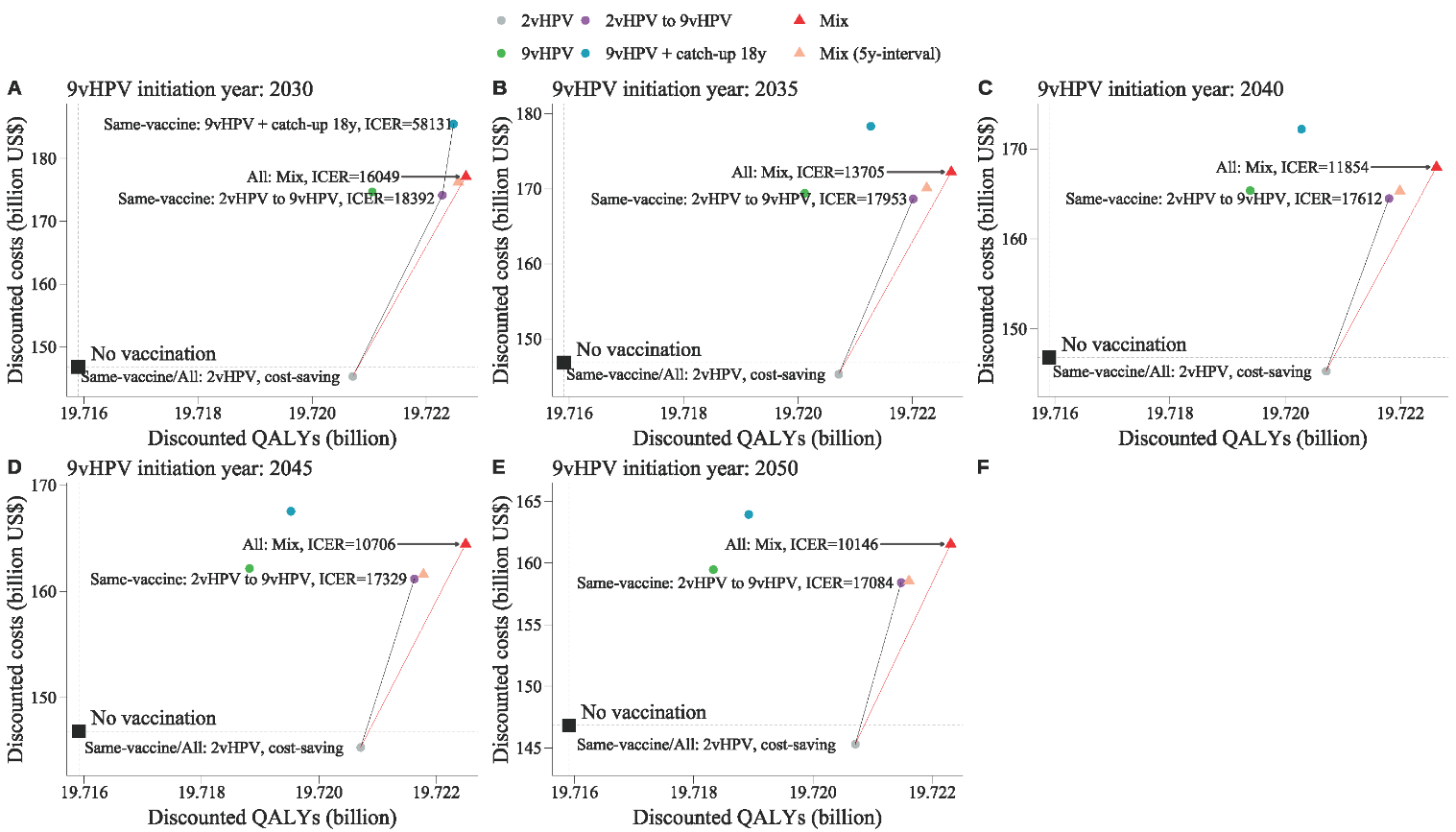
**

**Figure S11. Cost-effectiveness for different vaccination strategies under status quo screening assuming current vaccine prices**

Abbreviations: HPV, Human Papillomavirus; 9vHPV, nonavalent HPV vaccine; QALY, quality-adjusted life-year.

The black lines presented the cost-effectiveness frontier in same-vaccine strategies. The red lines presented the cost-effectiveness frontier in all same-vaccine and mixed-vaccine strategies.

**
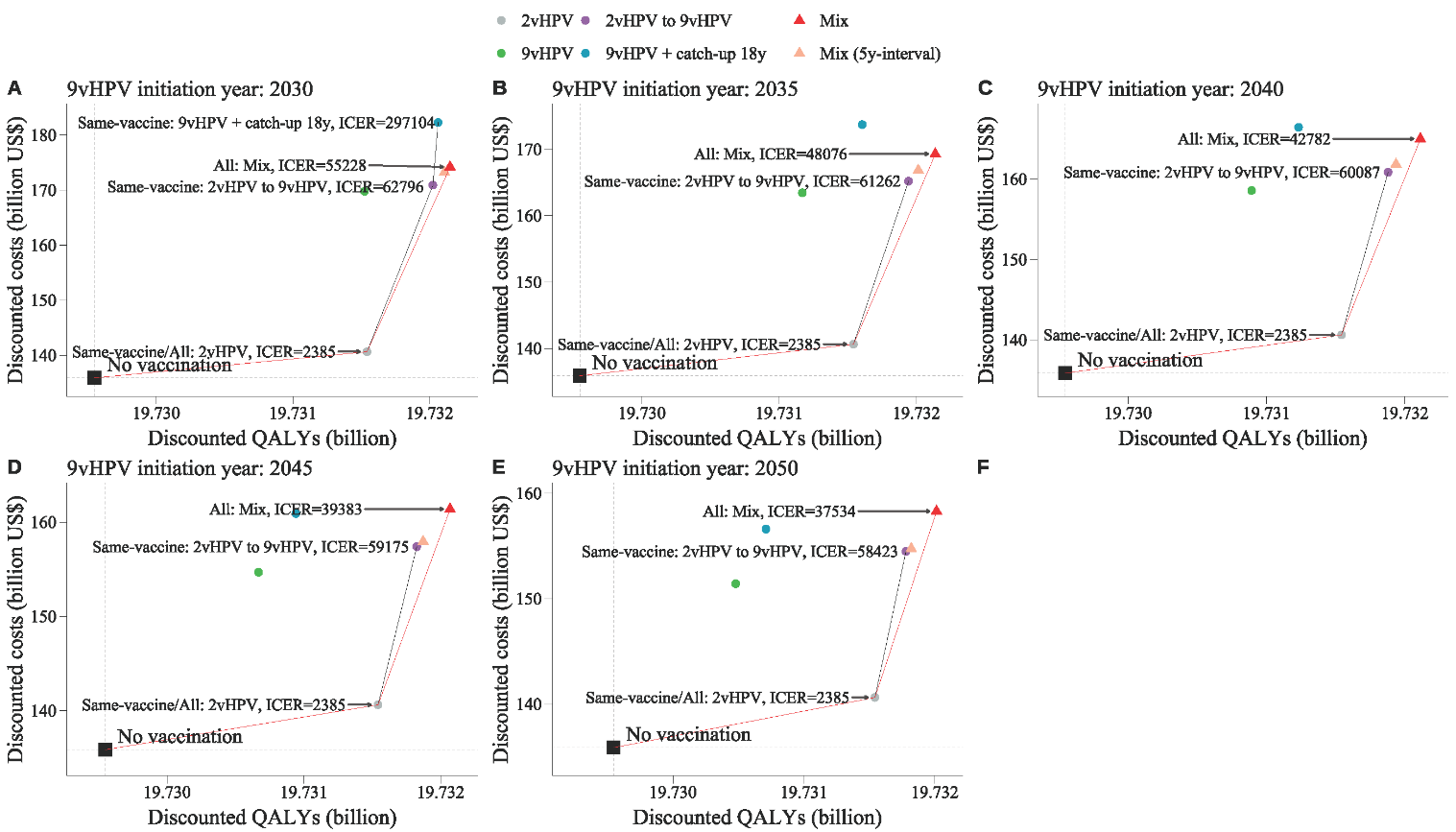
**

**Figure S12. Cost-effectiveness for different vaccination strategies under improved HPV-based screening assuming current vaccine prices**

Abbreviations: HPV, Human Papillomavirus; 9vHPV, nonavalent HPV vaccine; QALY, quality-adjusted life-year.

The black lines presented the cost-effectiveness frontier of same-vaccine strategies. The red lines presented the cost-effectiveness frontier of all same-vaccine and mixed-vaccine strategies.


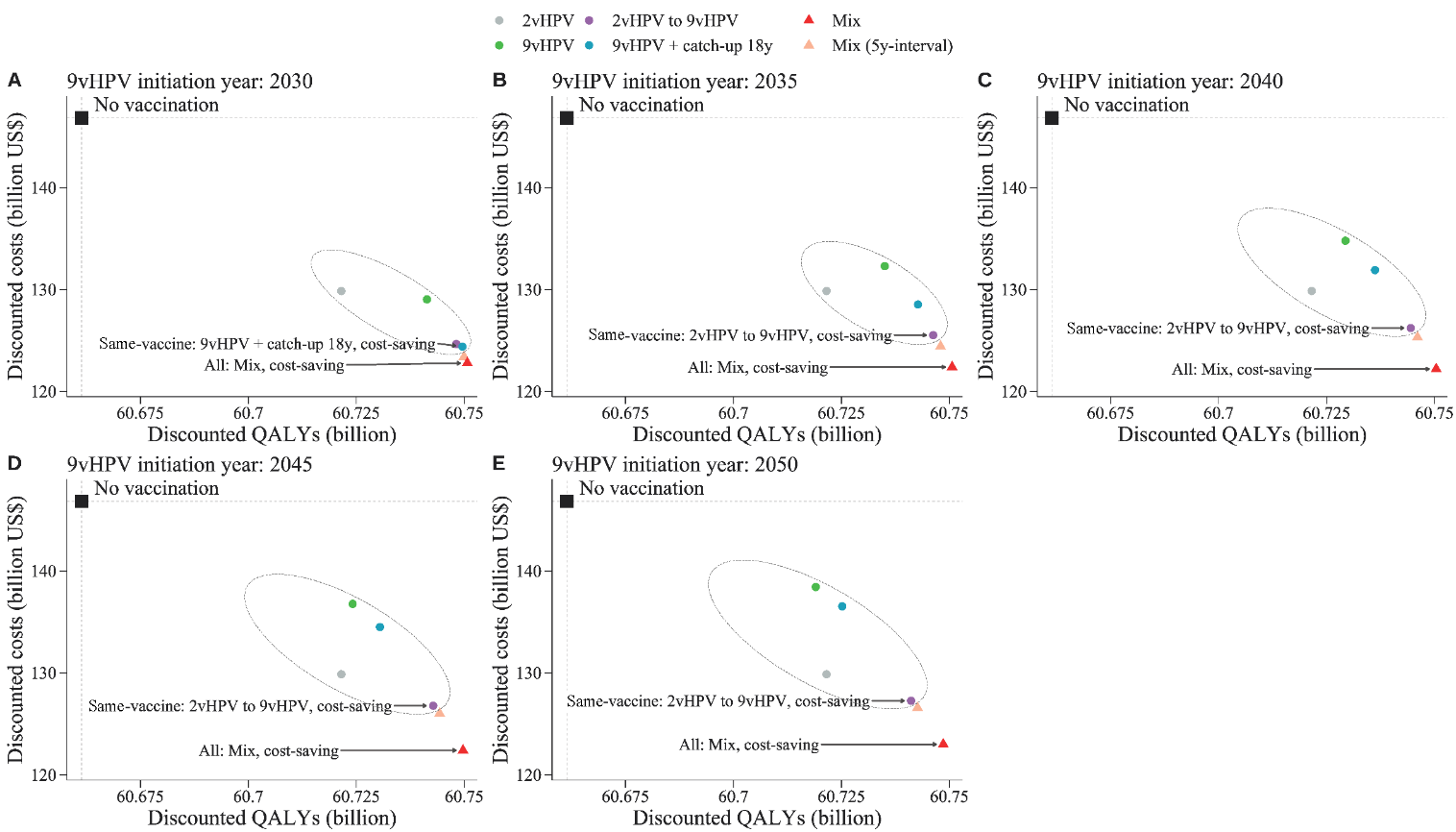


**Figure S13. Cost-effectiveness for different vaccination strategies under status quo screening using 3% and 0% discounting for costs and QALYs**

Abbreviations: HPV, Human Papillomavirus; 9vHPV, nonavalent HPV vaccine; QALY, quality-adjusted life-year.

The black squares indicate the reference strategy of no vaccination. The circles inside dashed ovals indicate the same-vaccine strategies, while the triangles indicate the mixed-vaccine strategies. The “mix” strategy represents mixed two-dose schedule (first dose of 2vHPV vaccine targeting girls aged 12 and a second dose of 9vHPV vaccine in the 9vHPV initiation year) from 2023, and switching to two-dose routine 9vHPV vaccination when 9vHPV vaccine is available for national vaccination. The “mix (5y-interval)” represents the strategy that adds the restriction of a 5-year maximum interval between two doses based on the “mix” strategy. The strategies in the upper left quadrant are dominated by the strategies in the lower right quadrant. The labels represent the dominant vaccination strategy in the in the same-vaccine strategies and all strategies (including same-vaccine and mixed-vaccine strategies), respectively. Because the dominant strategies are cost-saving, the ICERs are negative and not shown.


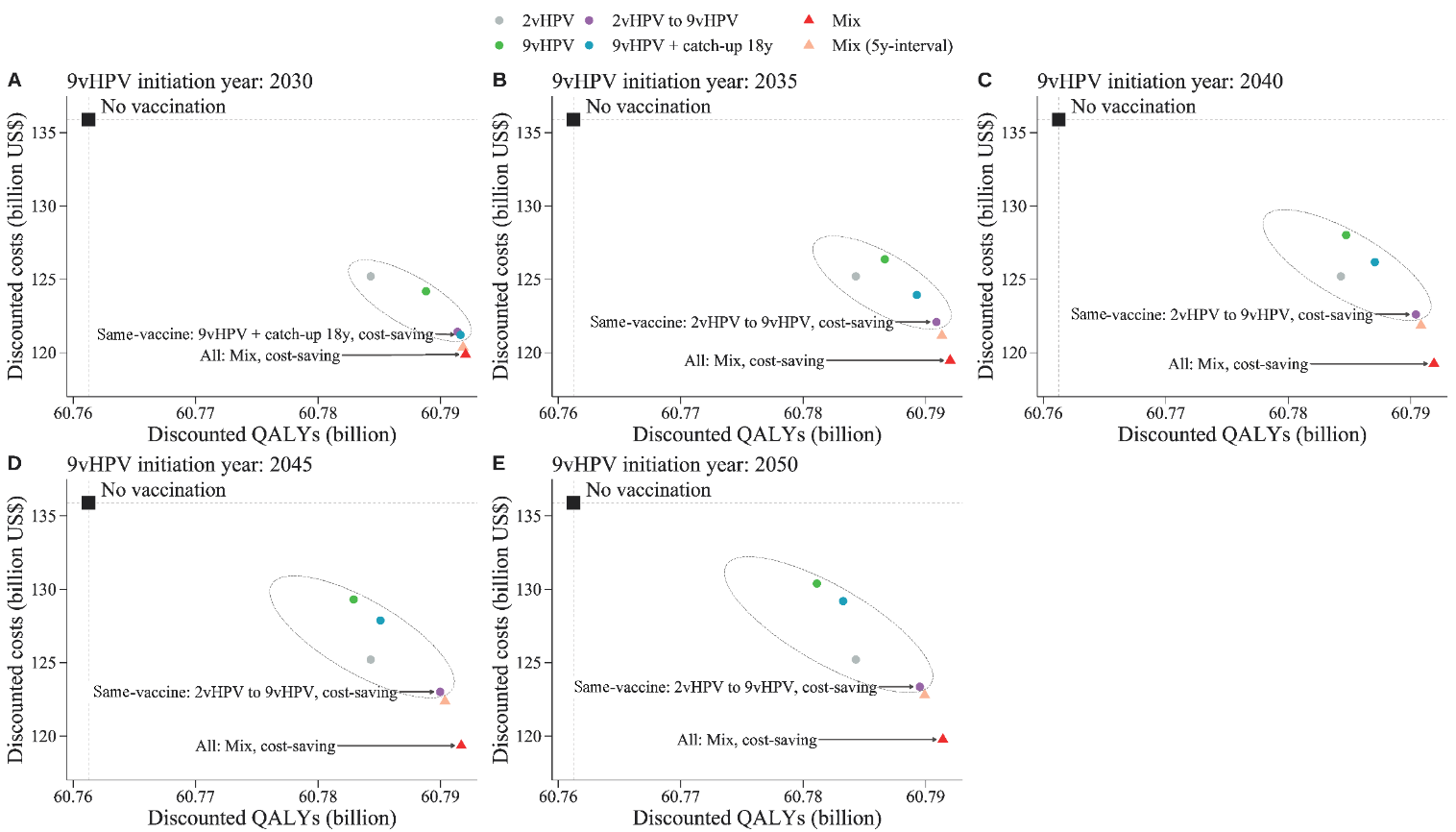


**Figure S14. Cost-effectiveness for different vaccination strategies under improved HPV-based screening using 3% and 0% discounting for costs and QALYs**

Abbreviations: HPV, Human Papillomavirus; 9vHPV, nonavalent HPV vaccine; QALY, quality-adjusted life-year.

The black squares indicate the reference strategy of no vaccination. The circles inside dashed ovals indicate the same-vaccine strategies, while the triangles indicate the mixed-vaccine strategies. The “mix” strategy represents mixed two-dose schedule (first dose of 2vHPV vaccine targeting girls aged 12 and a second dose of 9vHPV vaccine in the 9vHPV initiation year) from 2023, and switching to two-dose routine 9vHPV vaccination when 9vHPV vaccine is available for national vaccination. The “mix (5y-interval)” represents the strategy that adds the restriction of a 5-year maximum interval between two doses based on the “mix” strategy. The strategies in the upper left quadrant are dominated by the strategies in the lower right quadrant. The labels represent the dominant vaccination strategy in the same-vaccine strategies and all strategies (including same-vaccine and mixed-vaccine strategies), respectively. Because the dominant strategies are cost-saving, the ICERs are negative and not shown.

**Table S4. Estimated cervical cancer elimination year of different vaccination strategies by the 9vHPV initiation year, using World Female Population 2015**

| 9vHPV initiation year | Projected elimination year | | | | | |
| --- | --- | --- | --- | --- | --- | --- |
|  | Maintaining 2vHPV | Waiting for 9vHPV | 2vHPV to 9vHPV | 9vHPV+catch-up | Mixed-vaccine strategy (no interval restriction) | Mixed-vaccine strategy (5y-interval) |
| **Status quo screening** | | | | | | |
| 2030 | - | - | - | - | - | - |
| 2035 | - | - | - | - | - | - |
| 2040 | - | - | - | - | - | - |
| 2045 | - | - | - | - | - | - |
| 2050 | - | - | - | - | - | - |
| **Improved HPV-based screening** | | | | | | |
| 2030 | 2076 | 2069 | 2065 | 2062 | 2061 | 2063 |
| 2035 | 2076 | 2073 | 2066 | 2068 | 2061 | 2065 |
| 2040 | 2076 | 2078 | 2068 | 2073 | 2061 | 2066 |
| 2045 | 2076 | 2084 | 2070 | 2077 | 2062 | 2068 |
| 2050 | 2076 | 2089 | 2071 | 2083 | 2064 | 2070 |

Abbreviations: HPV, Human Papillomavirus; 2vHPV, bivalent HPV vaccine; 9vHPV, nonavalent HPV vaccine.

“-” indicates that cervical cancer will not be eliminated by 2100 by adopting this strategy. The improved HPV-based screening scenario represents switching to 5-yearly HPV-based screening in 2022, with linearly increasing age-specific uptake from status quo in 2021 to 70% in 2030, followed by a 1% increase every year till 90% is reached. Cervical cancer incidence was age standardised to the World Female Population 2015 (age 0-99 years).

**

**

**Figure S15. Deterministic sensitivity analysis for switching from 2vHPV to 9vHPV vaccine vs. maintaining 2vHPV vaccine under status quo screening**

Abbreviations: HPV, Human Papillomavirus; 9vHPV, nonavalent HPV vaccine; LBC, liquid-based cytology; CIN, cervical intraepithelial neoplasia; CC, cervical cancer; ICER, incremental cost-effectiveness ratio; QALY, quality-adjusted life-year.

**

**

**Figure S16. Deterministic sensitivity analysis for switching from 2vHPV to 9vHPV vaccine vs. maintaining 2vHPV vaccine under** **improved HPV-based screening**

Abbreviations: HPV, Human Papillomavirus; 9vHPV, nonavalent HPV vaccine; LBC, liquid-based cytology; CIN, cervical intraepithelial neoplasia; CC, cervical cancer; ICER, incremental cost-effectiveness ratio; QALY, quality-adjusted life-year.

**

**

**Figure S17. Deterministic sensitivity analysis for mixed-vaccine strategy (no interval restriction) vs. switching from 2vHPV to 9vHPV vaccine under status quo screening**

Abbreviations: HPV, Human Papillomavirus; 9vHPV, nonavalent HPV vaccine; LBC, liquid-based cytology; CIN, cervical intraepithelial neoplasia; CC, cervical cancer; ICER, incremental cost-effectiveness ratio; QALY, quality-adjusted life-year.

**

**

**Figure S18. Deterministic sensitivity analysis for mixed-vaccine strategy (no interval restriction) vs.** **switching from 2vHPV to 9vHPV vaccine under improved HPV-based screening**

Abbreviations: HPV, Human Papillomavirus; 9vHPV, nonavalent HPV vaccine; LBC, liquid-based cytology; CIN, cervical intraepithelial neoplasia; CC, cervical cancer; ICER, incremental cost-effectiveness ratio; QALY, quality-adjusted life-year.


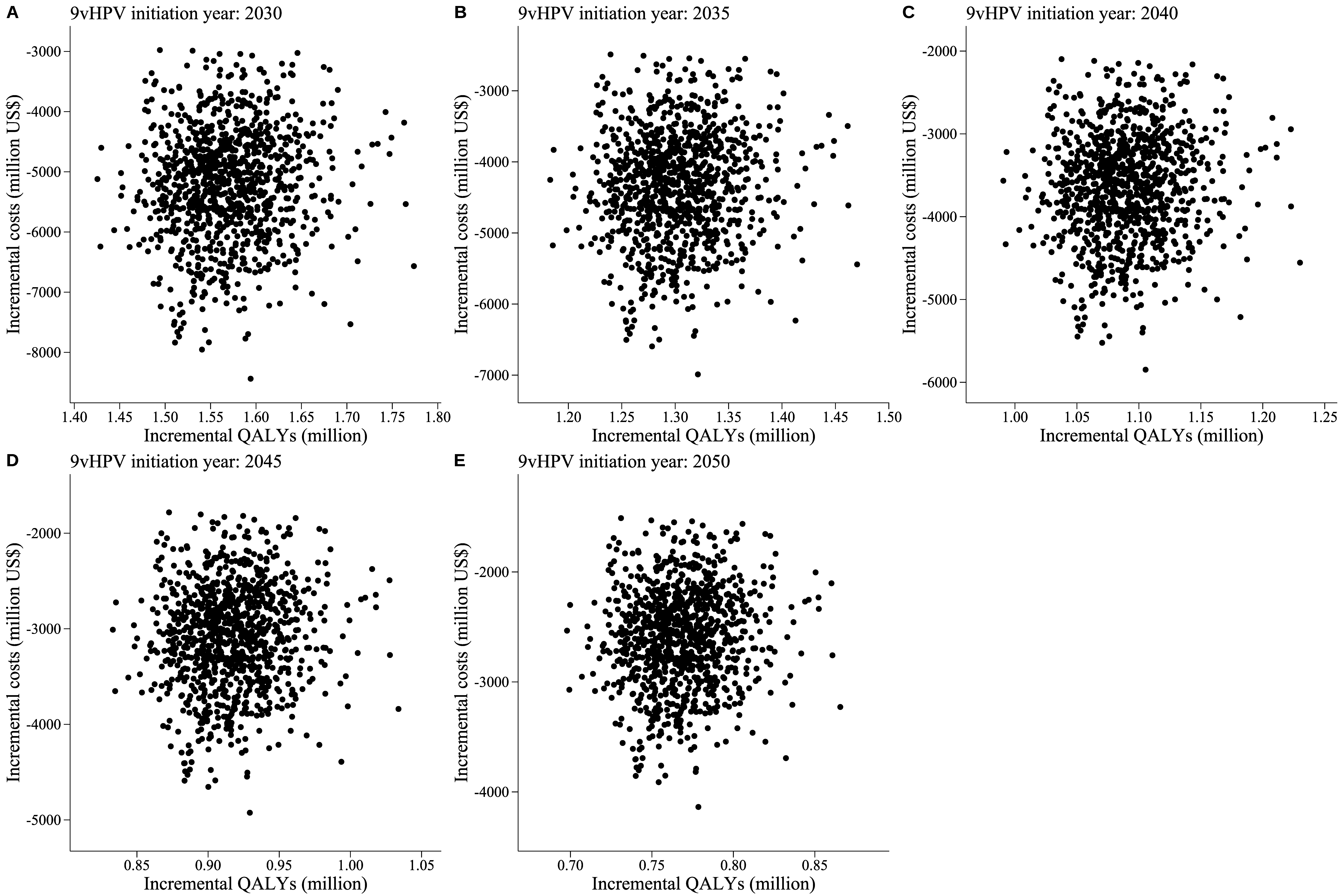


**Figure S19. Probabilistic sensitivity analyses for switching from 2vHPV to 9vHPV vaccine vs. maintaining 2vHPV vaccine under status quo screening**

Abbreviations: HPV, Human Papillomavirus; 9vHPV, nonavalent HPV vaccine; QALY, quality-adjusted life-year.


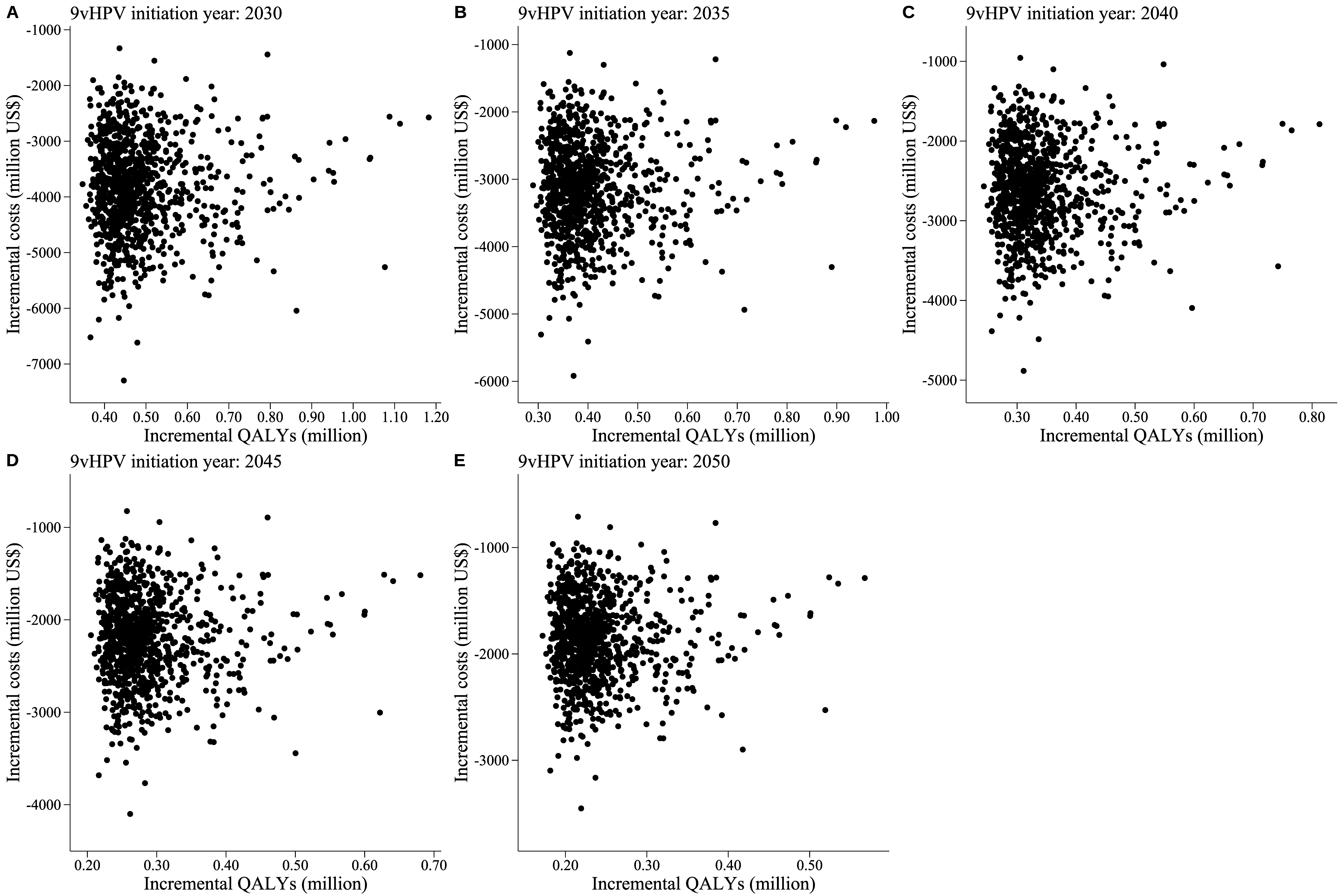


**Figure S20. Probabilistic sensitivity analyses for switching from 2vHPV to 9vHPV vaccine vs. maintaining 2vHPV vaccine under improved HPV-based screening**

Abbreviations: HPV, Human Papillomavirus; 9vHPV, nonavalent HPV vaccine; QALY, quality-adjusted life-year.


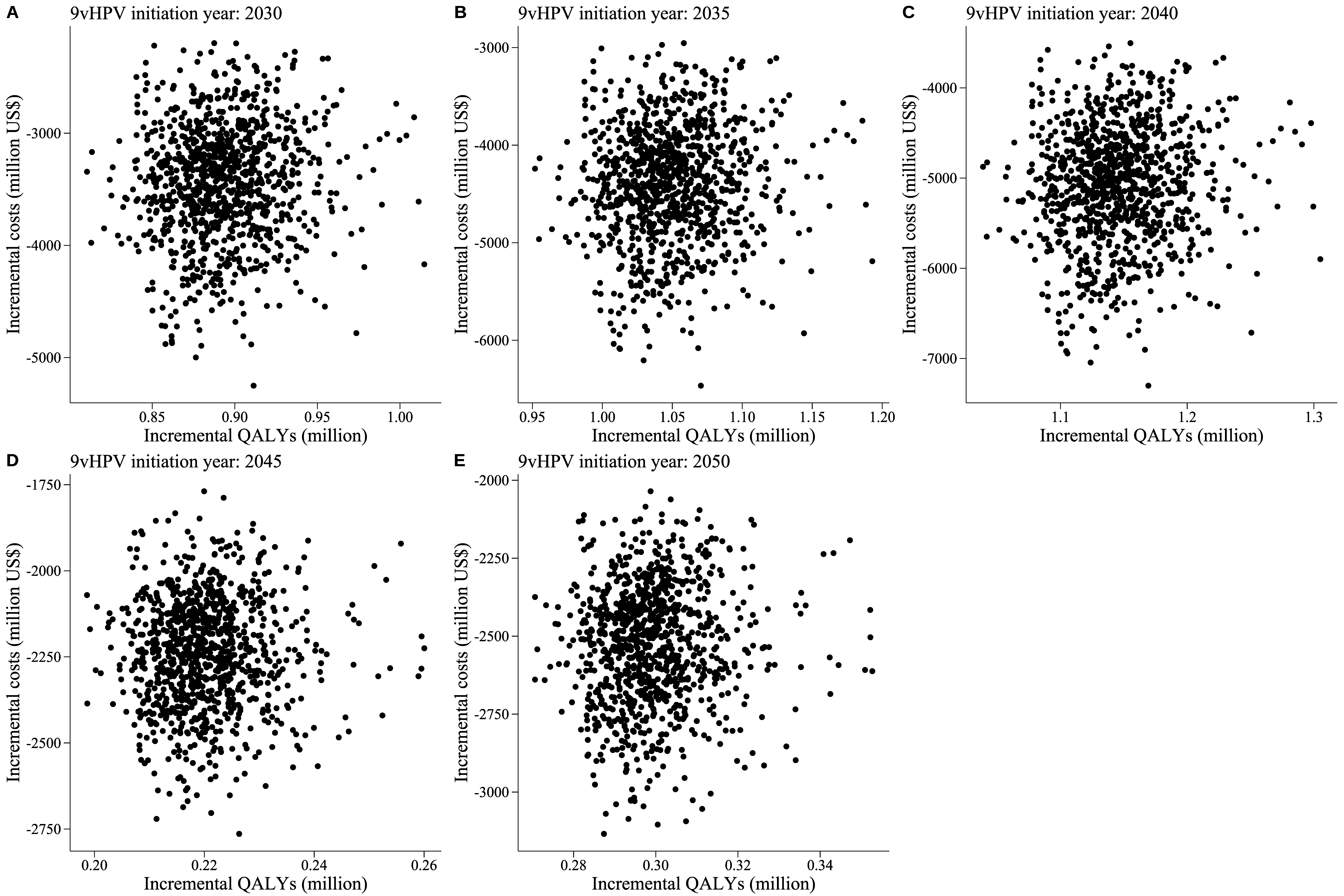


**Figure S21. Probabilistic sensitivity analyses for mixed-vaccine strategy (no interval restriction) vs. switching from 2vHPV to 9vHPV vaccine under status quo screening**

Abbreviations: HPV, Human Papillomavirus; 9vHPV, nonavalent HPV vaccine; QALY, quality-adjusted life-year.


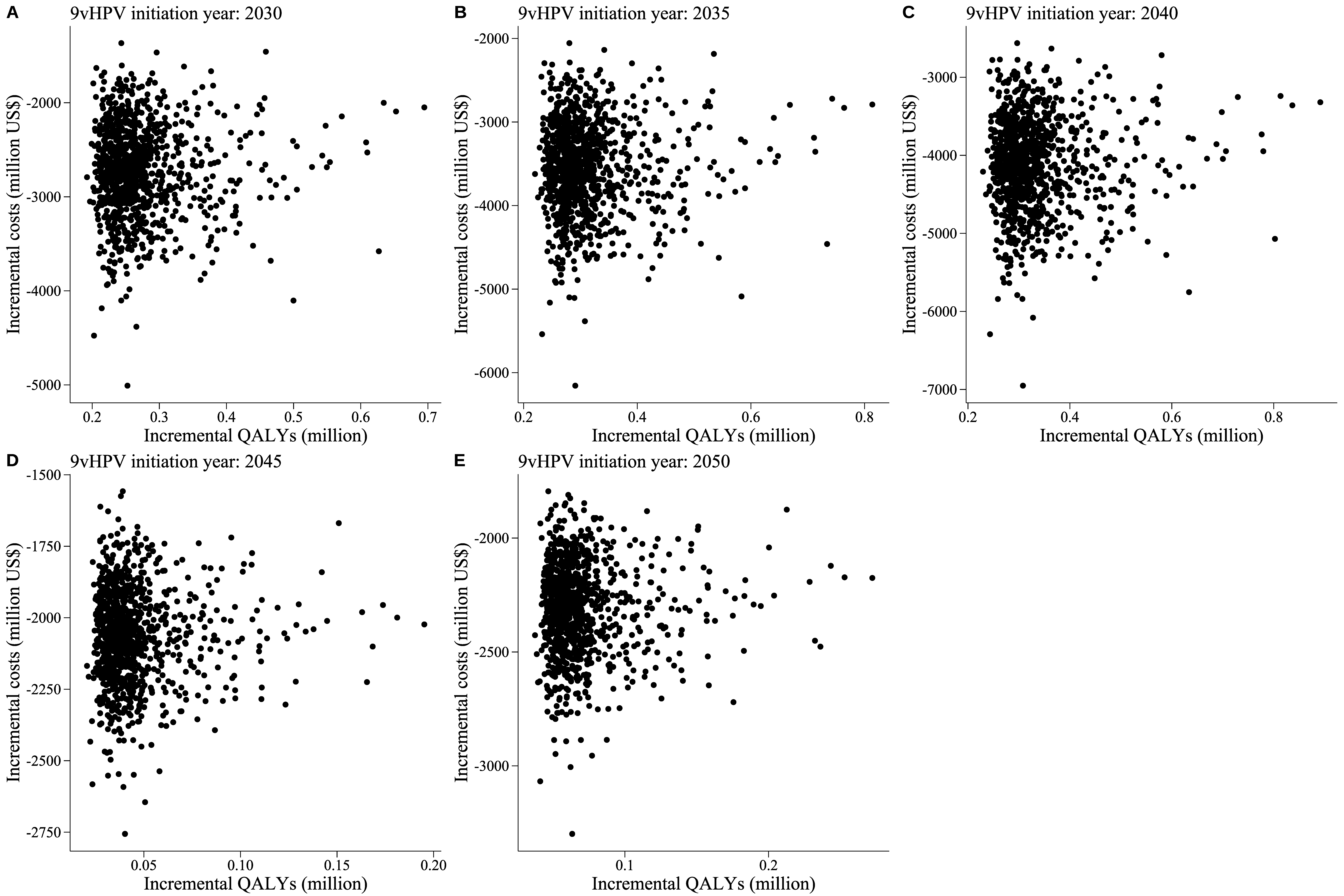


**Figure S22. Probabilistic sensitivity analyses for mixed-vaccine strategy (no interval restriction) vs. switching from 2vHPV to 9vHPV vaccine under improved HPV-based screening**

Abbreviations: HPV, Human Papillomavirus; 9vHPV, nonavalent HPV vaccine; QALY, quality-adjusted life-year.

Reference:

1. Xia C, Hu S, Xu X, et al. Projections up to 2100 and a budget optimisation strategy towards cervical cancer elimination in China: a modelling study. *The Lancet Public health* 2019; **4**(9): e462-e72.

2. Xia C, Xu X, Zhao X, et al. Effectiveness and cost-effectiveness of eliminating cervical cancer through a tailored optimal pathway: a modeling study. *BMC medicine* 2021; **19**(1): 62.

3. Pan American Health Organization. PAHO Revolving Fund Vaccine Prices for 2022. https://www.paho.org/en/documents/paho-revolving-fund-vaccine-prices-2022 (accessed June 1, 2022).

4. World Bank. World Bank Country and Lending Groups. 2022. https://datahelpdesk.worldbank.org/knowledgebase/articles/906519-world-bank-country-and-lending-groups (accessed June 1, 2022).

5. Chen H, Zhao X, Hu S, et al. Estimation of economic burden throughout course of cervical squamous intraepithelial lesion and cervical cancer in China: A nationwide multicenter cross-sectional study. *Chinese journal of cancer research = Chung-kuo yen cheng yen chiu* 2023; **35**(6): 675-85.

6. Canfell K, Barnabas R, Patnick J, Beral V. The predicted effect of changes in cervical screening practice in the UK: results from a modelling study. *British journal of cancer* 2004; **91**(3): 530-6.

7. Goldie SJ, Grima D, Kohli M, Wright TC, Weinstein M, Franco E. A comprehensive natural history model of HPV infection and cervical cancer to estimate the clinical impact of a prophylactic HPV-16/18 vaccine. *International journal of cancer* 2003; **106**(6): 896-904.

8. Haeussler K, den Hout AV, Baio G. A dynamic Bayesian Markov model for health economic evaluations of interventions in infectious disease. *BMC medical research methodology* 2018; **18**(1): 82.

9. Liu YJ, Zhang Q, Hu SY, Zhao FH. Effect of vaccination age on cost-effectiveness of human papillomavirus vaccination against cervical cancer in China. *BMC cancer* 2016; **16**: 164.

10. Haeussler K, Marcellusi A, Mennini FS, et al. Cost-Effectiveness Analysis of Universal Human Papillomavirus Vaccination Using a Dynamic Bayesian Methodology: The BEST II Study. *Value in health : the journal of the International Society for Pharmacoeconomics and Outcomes Research* 2015; **18**(8): 956-68.

11. Myers ER, McCrory DC, Nanda K, Bastian L, Matchar DB. Mathematical model for the natural history of human papillomavirus infection and cervical carcinogenesis. *American journal of epidemiology* 2000; **151**(12): 1158-71.

12. Yokoyama M, Iwasaka T, Nagata C, et al. Prognostic factors associated with the clinical outcome of cervical intraepithelial neoplasia: a cohort study in Japan. *Cancer letters* 2003; **192**(2): 171-9.

13. Sawaya GF, Sanstead E, Alarid-Escudero F, et al. Estimated Quality of Life and Economic Outcomes Associated With 12 Cervical Cancer Screening Strategies: A Cost-effectiveness Analysis. *JAMA internal medicine* 2019; **179**(7): 867-78.

14. Johnson HC, Elfström KM, Edmunds WJ. Inference of type-specific HPV transmissibility, progression and clearance rates: a mathematical modelling approach. *PloS one* 2012; **7**(11): e49614.

15. Abbas KM, van Zandvoort K, Brisson M, Jit M. Effects of updated demography, disability weights, and cervical cancer burden on estimates of human papillomavirus vaccination impact at the global, regional, and national levels: a PRIME modelling study. *The Lancet Global health* 2020; **8**(4): e536-e44.

16. Brisson M, Kim JJ, Canfell K, et al. Impact of HPV vaccination and cervical screening on cervical cancer elimination: a comparative modelling analysis in 78 low-income and lower-middle-income countries. *Lancet (London, England)* 2020; **395**(10224): 575-90.

17. Canfell K, Kim JJ, Brisson M, et al. Mortality impact of achieving WHO cervical cancer elimination targets: a comparative modelling analysis in 78 low-income and lower-middle-income countries. *Lancet (London, England)* 2020; **395**(10224): 591-603.

18. Cuzick J, Clavel C, Petry KU, et al. Overview of the European and North American studies on HPV testing in primary cervical cancer screening. *International journal of cancer* 2006; **119**(5): 1095-101.

19. Yu W, Lu M, Wang H, et al. Routine immunization services costs and financing in China, 2015. *Vaccine* 2018; **36**(21): 3041-7.

20. van Rosmalen J, de Kok IM, van Ballegooijen M. Cost-effectiveness of cervical cancer screening: cytology versus human papillomavirus DNA testing. *BJOG : an international journal of obstetrics and gynaecology* 2012; **119**(6): 699-709.

21. Zhao ZM, Pan XF, Lv SH, et al. Quality of life in women with cervical precursor lesions and cancer: a prospective, 6-month, hospital-based study in China. *Chinese journal of cancer* 2014; **33**(7): 339-45.

22. WHO. Global strategy to accelerate the elimination of cervical cancer as a public health problem. 2020. https://www.who.int/publications/i/item/9789240014107 (accessed June 1, 2022).

23. Burger EA, Smith MA, Killen J, et al. Projected time to elimination of cervical cancer in the USA: a comparative modelling study. *The Lancet Public health* 2020; **5**(4): e213-e22.

24. Haacker M, Hallett TB, Atun R. On discount rates for economic evaluations in global health. *Health policy and planning* 2020; **35**(1): 107-14.

25. National Bureau of Statistics of China. National Data. http://data.stats.gov.cn/english/ (accessed June 1, 2022).

26. National Bureau of Statistics of China. China Population and Employment Statistics Yearbook 2016. Beijing: China Statistics Press; 2017.

27. National Health and Family Planning Commission. 2016 China Health Statistics Yearbook. Beijing: Peking Union Medical College Press; 2016.

28. He J. 2018 China Cancer Registry Annual Report. Beijing, 2019.

29. United Nations Department of Economic and Social Affairs Population Division. World Population Prospects: The 2017 Revision. 2017. https://population.un.org/wpp/Download/Standard/Fertility/ (accessed June 1, 2022).

30. United Nations Department of Economic and Social Affairs Population Division. World Urbanization Prospects: The 2018 Revision. 2018. https://population.un.org/wup/Download/ (accessed June 1, 2022).

31. Gu CL, Guan WH, Liu HL. Chinese urbanization 2050: SD modeling and process simulation. *Science China Earth Sciences* 2017.

32. UN Development Programme China, Development Research Center of the State Council of China. China national human development report 2016: social innovation for inclusive human development. 2016. http://hdr.undp.org/sites/default/files/2016_human_development_report.pdf (accessed June 1, 2022).

33. Bao H, Zhang L, Wang L, et al. Significant variations in the cervical cancer screening rate in China by individual-level and geographical measures of socioeconomic status: a multilevel model analysis of a nationally representative survey dataset. *Cancer medicine* 2018; **7**(5): 2089-100.

34. Bao H, Wang L, Wang L, et al. Study on the coverage of cervical and breast cancer screening among women aged 35-69 years and related impact of socioeconomic factors in China, 2013. *Chinese Journal of Epidemiology* 2018; 39(2):208-212.
